# Evaluating the 2014 sugar-sweetened beverage tax in Chile: An observational study in urban areas

**DOI:** 10.1371/journal.pmed.1002596

**Published:** 2018-07-03

**Authors:** Ryota Nakamura, Andrew J. Mirelman, Cristóbal Cuadrado, Nicolas Silva-Illanes, Jocelyn Dunstan, Marc Suhrcke

**Affiliations:** 1 Centre for Health Economics, University of York, York, United Kingdom; 2 Hitotsubashi Institute for Advanced Study, Hitotsubashi University, Tokyo, Japan; 3 School of Public Health, University of Chile, Santiago, Chile; 4 ICBM, Faculty of Medicine, University of Chile, Santiago, Chile; 5 Luxembourg Institute for Socio-economic Research, Esch-sur-Alzette, Luxembourg; University of Cambridge, UNITED KINGDOM

## Abstract

**Background:**

In October 2014, Chile implemented a tax modification on sugar-sweetened beverages (SSBs) called the Impuesto Adicional a las Bebidas Analcohólicas (IABA). The design of the tax was unique, increasing the tax on soft drinks above 6.25 grams of added sugar per 100 mL and decreasing the tax for those below this threshold.

**Methods and findings:**

This study evaluates Chile’s SSB tax, which was announced in March 2014 and implemented in October 2014. We used household-level grocery-purchasing data from 2011 to 2015 for 2,836 households living in cities representative of the urban population of Chile. We employed a fixed-effects econometric approach and estimated the before–after change in purchasing of SSBs controlling for seasonality, general time trend, temperature, and economic fluctuations as well as time-invariant household characteristics. Results showed significant changes in purchasing for the statistically preferred model: while there was a barely significant decrease in the volume of all soft drinks, there was a highly significant decrease in the monthly purchased volume of the higher-taxed, sugary soft drinks by 21.6%. The direction of this reduction was robust to different empirical modelling approaches, but the statistical significance and the magnitude of the changes varied considerably. The reduction in soft drink purchasing was most evident amongst higher socioeconomic groups and higher pretax purchasers of sugary soft drinks. There was no systematic, robust pattern in the estimates by household obesity status. After tax implementation, the purchase prices of soft drinks decreased for the items for which the tax rate was reduced, but they remained unchanged for sugary items, for which the tax was increased. However, the purchase prices increased for sugary soft drinks at the time of the policy announcement. The main limitations include a lack of a randomised design, limiting the extent of causal inference possible, and the focus on purchasing data rather than consumption or health outcomes.

**Conclusions:**

The results of subgroup analyses suggest that the policy may have been partially effective, though not necessarily in ways that are likely to reduce socioeconomic inequalities in diet-related health. It remains unclear whether the policy has had a major, overall population-level impact. Additionally, because the present study examined purchasing of soft drinks for only 1 year, a longer-term evaluation—ideally including an assessment of consumption and health impacts—should be conducted in future research.

**Trial registration:**

ClinicalTrials.gov Identifier: NCT02926001

## Abstract 2

Ryota Nakamura and colleagues present findings from an observational study of sugar-sweetened beverage purchases in Chile after a tax change.

## Author summary

**Why was this study done?:**

- Chile and other countries in the world are facing a major challenge to curb rising rates of obesity and diet-related ill health, often particularly among lower socioeconomic groups.
- Fiscal policy has the potential to influence consumption patterns towards healthier options (and raise government revenue for welfare-enhancing purposes), thereby contributing to the prevention of chronic diseases and to the reduction of the associated economic costs.
- Most existing evidence on the effects of fiscal incentives to promote diet-related health relies on small-scale experiments or hypothetical simulations using price elasticity estimated from observational data, largely suggesting that increasing the price of products such as sugar-sweetened beverages (SSBs) would reduce purchasing or consumption. There is, however, a scarcity of empirical evaluations of actual diet-related fiscal interventions, and it is still uncertain whether these can be effective in improving population diet and health in a ‘real world’ context.

**What did the researchers do and find?:**

- This study evaluates the tax policy applied to SSBs in Chile. The ‘Impuesto Adicional a las Bebidas Analcohólicas’ (IABA; or ‘additional tax on nonalcoholic drinks’), which was announced in March 2014 and implemented in October 2014. The IABA policy targeted any nonalcoholic beverages to which colorants, flavourings, or sweeteners have been added. For beverages with an added sugar concentration of 6.25 grams per 100 mL (or equivalent proportion) or more, the tax was increased from 13% to 18%, while for those below this threshold, the tax was decreased from 13% to 10%, producing an 8% tax difference.
- We applied a series of econometric approaches to evaluate the effects of the announcement and implementation of the tax policy, using household-level grocery-purchasing data from Chile for 3 years pre- and 1 year post-tax implementation.
- We find that, despite the fact that the tax incentive is comparatively small, there are signs that purchasing of SSBs with higher sugar content has been reduced among high socioeconomic groups of the population.

**What do these findings mean?:**

- The results suggest that the Chilean tax policy may have been partially effective, though not necessarily in ways that are likely to reduce socioeconomic inequalities in diet-related health.
- Longer-term evaluations are needed to analyse the effect on SSB purchasing in the long run as well as on health outcomes.
- The evaluation did not involve a randomised design, therefore the degree to which we can interpret the results as truly causal will be limited.

## Introduction

Reducing the global burden of noncommunicable diseases (NCDs) has been widely recognised as a global health priority [1]. Diet-related health problems account for a large and growing share of the NCD burden worldwide, causing substantial human suffering and adverse economic consequences [2,3]. The situation is of particular concern in Latin America and the Caribbean—a region that has been predicted to reach the highest levels of overweight and obesity worldwide by 2030 [4].

The 2013 Global Burden of Disease estimates show that high body mass index (BMI) is the second most important risk factor in Chile [5]. As evidence from various Chilean surveys consistently suggests, obesity has been increasing steadily in children, adults, and pregnant women over the 2000–2010 period [6]. Evidence also indicates that it is those from lower socioeconomic backgrounds in Chile who are particularly prone to being overweight and obese [7,8]. In 2014, Chile topped the worldwide ranking of per capita daily sugar-sweetened beverage (SSB) consumption while also recording the highest growth rate of SSB consumption in the 2009–2014 period [9]. The Chilean National Food Consumption Survey (2010–2012) shows that the median consumption of SSBs, a factor that has been directly linked with child obesity, in Chilean children is close to 500 mL per day [10]. To put this into perspective, such consumption levels are only matched by the highest-consuming 5% of United States children [11].

Fiscal policies have the potential to promote healthier consumption patterns, thereby contributing to the prevention of chronic diseases and their associated economic costs. They may also lead to increased government revenue that could, in turn, be used for welfare-enhancing purposes. The current evidence base, however, is decidedly mixed as to what the expected effects of a fiscal incentive will be [12,13]. The majority of the evidence rests on the modelling of hypothetical policy scenarios rather than on the evaluation of real-life policy reforms. A small number of countries have recently implemented taxes on certain types of ‘unhealthy’ foods and/or beverages, as characterised by their high sugar, fat, or salt content. Evaluation evidence from Mexico [14], Denmark [15], Hungary [16], and from Berkeley, California [17] does highlight some early success in these cases.

Since 1960, Chile has had a tax on nonalcoholic drinks called the Impuesto Adicional a las Bebidas Analcohólicas (IABA; or ‘additional tax on soft drinks’) [18,19]. This is an ad valorem tax, and the tax rate has been fixed at 13% since 1976, without any formal impact evaluation. In March 2014, the Parliament announced a modification of IABA. At that point, the plan was to focus on a tax increase (by 5%) for all soft drinks containing any added sugar. At a later point—but still ahead of the October 2014 implementation—it was decided to reduce the tax rate by 3% for soft drinks containing low sugar levels. In October 2014, Chile implemented the tax modification. The tax affects any nonalcoholic beverages, or soft drinks, to which colourants, flavourings, or sweeteners have been added. For beverages above an added sugar concentration of at least 6.25 grams per 100 mL, the tax was increased from 13% to 18%, while for those below this threshold, the tax was decreased from 13% to 10%, effectively creating an 8% tax difference between these beverage groups. This means that, if the tax was fully transmitted to the consumer price, the price of a typical, 500-mL sugary carbonated drink would increase from about 500 to 525 pesos. For the less sugary drink of the same size, the price would decrease from about 500 to 485 pesos. For the sugary drink item, the magnitude of price change in Chile is comparable to or slightly smaller than the price change that would be created by a complete pass-through of the tax that was implemented in Berkeley (0.01 US dollar per ounce, equivalent to an increase in price from 500 to 563 Chilean pesos based on 2014 Purchasing Power Parity) [17] and in Mexico (1 Mexican peso per litre, equivalent to an increase in price from 500 to 524 Chilean pesos) [20]. For other beverages without flavour, sweeteners, or colourants (e.g., plain mineral water), there was no tax prior to the reform, and no additional taxation was introduced. These tax rates were applied in addition to the existing value-added tax (VAT) of 19%, and no other major policies were implemented around the same time that would influence purchasing and consumption of soft drinks.

There are several ways in which the nature of the tax policy in Chile is different from tax policies on soft drinks implemented elsewhere. For example, in Berkeley and Mexico, the size of the tax amount increases linearly with the volume of the relevant sugary drink (as defined in the policy). The Chilean tax, on the other hand, set a threshold of sugar content and applied either a tax rate increase or decrease to products above or below the threshold. The simultaneous tax increase and tax decrease makes Chile a unique case.

In this paper, we undertake an evaluation of Chile’s IABA tax to assess whether household purchasing of soft drinks have been reduced after the policy’s announcement and implementation. Our analysis protocol was published at www.ClinicalTrials.gov (Identifier: NCT02926001) before the study commenced [21]. We set out to answer the following research questions:

1. Were the announcement and implementation (and/or the announcement) of the IABA tax policy associated with a change in the volume of soft drinks purchased and the amount of sugar from soft drinks purchased?
2. Was the policy associated with a change in the prices of soft drinks that consumers paid?
3. Was the policy associated with a change in the shopping patterns of consumers, including the frequency of purchases of soft drinks and the use of price promotions?
4. Were there differential changes in outcomes of interest by key household characteristics, i.e., socioeconomic status (SES), BMI, and the pretax purchasing of soft drinks?

## Methods

### Data

We used household-level food-purchasing data collected by a leading commercial research company (Kantar WorldPanel) for the time period of January 2011 to December 2015 (https://www.kantarworldpanel.com/cl/; contact: María Paz Román: mariapaz.roman@kantarworldpanel.com). The data are based on households’ weekly purchases of ‘fast-moving consumer goods’—i.e., beverages, grocery (except for meats, fruits, and vegetables), dairy, household cleaners, and personal care items—from cities with more than 20,000 inhabitants, representing 74% of the urban population. The survey aimed at collecting information from 2,000 households, but due to replacements of households during the survey period, the raw data contain a total of 2,836 households. The data include detailed transaction records of the take-home purchases, and for the present study, we focused on the category of nonalcoholic beverages (excluding coffee, tea, and dairy products).

The data also include information on relevant household characteristics such as SES, region of residence, and BMI of all household members. SES is determined by a Chile-specific measure that combines occupation and level of education of the household head and the size of a household’s property [22]. BMI is self-reported and recorded once per year.

The Kantar WorldPanel used the 2002 national census survey and other sources to define the target population for sampling. A stratified random sampling method, coupled with the Random Iterative Method weighting [23], was then performed by the data company. The sample is representative with respect to the age of the main shopper, household SES, and the region of residence (North, Valparaiso Region, Center South, Bio-Bio Region, and South).

Households who agreed to participate in the survey were asked by Kantar WorldPanel to keep information of purchased items in the form of till receipts, product packages with barcodes, and a daily log sheet of purchases. The log sheet served as a source for products without barcodes or when till receipts were not provided. Kantar WorldPanel employed field workers to visit the households each week to record the purchases using the collected information.

Observations for households were included by Kantar WorldPanel if a household provided satisfactory information in the first 2 months. The quality of data from each household was monitored continuously; when missing data, inaccurate reporting, and/or misreporting were detected, Kantar WorldPanel estimated purchases using information from other households in the same residential area. In rare cases, missing data were imputed using data from households with similar socioeconomic and/or demographic characteristics. When households dropped out of the survey due to an unwillingness to continue (e.g., health reasons), change of physical address, or poor compliance, then new households with similar characteristics were recruited to maintain the sample size and representativeness.

The replacement rate of households was about 15% per year. This is comparable, for example, with a national longitudinal survey of poverty in Chile (i.e., 10%–16% [La Encuesta Panel CASEN data]) and a major household survey in the US (i.e., 7.7%–14.8% [The Medical Expenditure Panel Survey]). During the survey at hand, each month approximately 3% of the households provided insufficient data. Those households were excluded from the database for that specific month (which is indicated in the data). If such a household consistently showed data quality problems, the household was replaced.

Because the Kantar WorldPanel data do not contain nutritional information for specific products, we complemented the product information in the data with information on sugar content for all products that account for the top 90% of sales of all soft drinks in the Kantar WorldPanel data. This information was collected from several sources, including a large, nationally representative survey [24], manufacturers’ documents and web pages, and national health authorities’ surveillance systems [25]. All soft drink products were coded into the following 3 categories based on the sugar content threshold set out in the IABA policy: (1) nontaxed products (nonsugary, nonflavoured, noncoloured products), (2) low-taxed products (containing equal to or less than 6.25 grams of sugar per 100 mL), and (3) high-taxed products (containing more than 6.25 grams of sugar per 100 mL). We calculated the monthly volume of purchased items for these 3 categories and hereafter refer to them as ‘no-tax’, ‘low-tax’, and ‘high-tax’.

We constructed a monthly, household-level panel dataset to analyse the impact of the IABA policy on the volume of soft drinks purchased. In addition, we constructed a product-level panel dataset at the level of stock-keeping unit (SKU) and used the average price of products in each month to examine the impact of the tax on prices paid by the households. It should be noted that the Kantar WorldPanel offers ‘transaction-based’ data, i.e., the price of a product is recorded only when a transaction is made. These data capture the prices that were actually paid by households rather than the prices that were posted in the stores. In other words, the price data capture an aspect of consumers’ behavioural response to the tax, namely, price-accepting behaviour. For example, if the average price paid for a product or product category has not changed in response to the tax, this could mean that the consumers did not accept the higher price, and so they may reduce purchases of soft drinks or buy items only when the price appears reasonable to them. This contrasts with previous analyses that have looked at the impact of a tax on prices that were posted in stores and thus limits the extent to which we can draw comparisons with studies of price pass-through [26].

Finally, we also linked the Kantar WorldPanel data with information for regional unemployment and temperature. For unemployment, we used the database provided by the Chilean National Institute of Statistics [27], and for temperature, data were obtained from the Centre for Climate Research and Resiliency [28].

### Analytic methods

In this study, we employed a quasi-experimental approach to evaluate the impact of the tax policy. There was no experimental manipulation of the tax rate; instead, the government decided to introduce the tax policy to reduce the high prevalence of obesity and related NCDs. Nonetheless, we regarded the policy implementation as an intervention that generated variations in the tax rate, and we estimated the impact of the policy under certain assumptions described below [29,30].

Because the tax policy was implemented nationwide at a single point in time, the entire Chilean population was exposed to the policy, and there are no direct comparison groups for the evaluation within Chile. Our empirical approach therefore relies on the time series variations before and after the tax implementation in October 2014.

For the analyses of volumes and prices of items purchased, we used a fixed-effects regression approach [31]. A key assumption of this approach is that, after controlling for households’ (time-invariant) unobserved characteristics and the general time trend and seasonality of the outcome variables, any remaining changes in purchasing of soft drinks in the post-tax period are attributable to the tax. If there are other factors that are time-varying and affect the outcome variables that are unaccounted for, then the estimated impacts will be biased.

We estimated the following linear model with household fixed effects:

$${logY}_{it}=\beta_{i}+\tau Post+{x_{it}}^{\prime }\varphi +f(t)+\delta_{month}+\epsilon_{it},$$ (1)

where logY_it_ is the log of per capita volume of items purchased by household *i* at period *t*. If a panel household did not show any record of purchasing of soft drinks for a given month, the outcome variable for the household was imputed as 0. The household fixed effect is represented by *β_i_*, which captures household time-invariant unobservable characteristics that may affect purchasing of soft drinks. ‘Post’ is an indicator variable taking a value of 1 if the purchase is made on or after 1 October 2014, and 0 otherwise. The vector **x**_*it*_ includes the temperature and unemployment rate for a household’s region of residence. Temperature controls for the variation of demand for soft drinks due to climate, whereas the unemployment rate controls for the variation of macroeconomic conditions. The term f(t) is a function to control for a general time trend. We used a flexible approach to polynomial modelling, and the Akaike Information Criterion (AIC) was employed to select the order of the polynomial with the best fit. In this study, we selected a polynomial of order 4, i.e., f(t) = α_1_t + α_2_t^2^ + α_3_t^3^ + α_4_t^4^ (where α_1_, α_2_, α_3_, and α_4_ are parameters and t is centered at October 2014, when the policy was implemented, taking a value of 0). The term δ_month_ represents the month of the year effects, which captures seasonality. Finally, ϵ_it_ is the idiosyncratic error term. The within-household impact of the tax policy is represented by the parameter τ.

To look at the change in the prices paid for items purchased by the consumers, we used a similar model specification but with SKU-level fixed effects. The model is given as the following:

$${logP}_{it}=\alpha_{i}+\theta Post+g(t)+\delta_{month}+\varepsilon_{it},$$ (2)

where log *P_it_* is the logged average price per unit volume of product *i* at period *t*. ‘Post’ is again the policy indicator variable, *α_i_* represents product fixed effects, capturing a product’s time-invariant unobservable characteristics, and g(t) is a polynomial to control for a general time trend, selected based on AIC (corresponding to f(t) in equation [1]). The idiosyncratic error is represented by ε_it_. In this model, the parameter θ captures the impact of the tax after it is introduced.

To explore the sensitivity of the results to other modelling approaches, we complemented the above fixed-effects regression approach with a difference-in-difference (DiD) approach. This approach uses previous time periods of the treated population as a comparison group and thus diverges from a standard DiD approach that uses 2 distinct groups. A similar methodology was employed in a previous evaluation of the impact of an SSB tax in Mexico [14]. For further details, see S1 Text.

### Outcome variables and subsample analyses

#### Differential changes by tax category and share of soft drink purchases

Given that the IABA tax policy increased the tax rate by 5% (from 13% to 18%) for soft drinks above the sugar threshold but reduced the tax rate by 3% for beverages below the sugar threshold, we conducted the analysis for ‘low-tax’ products and ‘high-tax‘ products separately. We also looked at trends in the separate ‘no-tax’ product category.

In an extension of our main analysis, we also ran the models with the outcome variable as the share of purchases of all soft drinks making up a specific tax category. This was done to analyse changes in the composition of the basket of all soft drinks that consumers purchased in a month.

#### Policy announcement

One possibility is that a change in purchasing of soft drinks may have occurred when the policy was announced at the end of March 2014, several months prior to implementation. Consumers and the beverage industry could have changed behaviours and practices solely due to the policy’s announcement—e.g., consumers could anticipate purchases in bulk to save on future price increases, and the industry could potentially increase the price prior to the actual implementation date. Therefore, the expected overall change in purchasing is ex ante ambiguous. We examined the announcement effect by running models (1) and (2) with the ‘Post’ variable set to April 2014, directly after the announcement date in late March.

#### Volume of sugar from soft drink items

A key objective of the policy was to reduce the amount of sugar purchased by reducing the purchases of high–sugar-content products. Because we collected information on the sugar content of all soft drinks, we estimated the association between the policy implementation and the volume of added sugar purchased from all soft drinks.

#### Shopping patterns

It is conceivable that, in response to the tax, the households might have decided to cut down on the frequency with which they purchased the higher-taxed soft drinks, and they may have also cut down on the volume of those products purchased in each shopping occasion in response to the higher price. It may also be that consumers increasingly targeted price promotions to cope with the expected price increase for the higher-taxed soft drinks. To examine this, we looked at changes in household shopping patterns in 2 ways. First, we analysed the number of days per month involving purchases of any soft drinks, and second, we explored whether the households used more price promotions in the post-tax compared to the pretax period.

#### Differential changes by socioeconomic group, BMI, and pretax purchased volume

We also investigated whether a change in purchasing was more pronounced for poorer groups than for wealthier ones. This was done by dividing the households into 3 socioeconomic groups and conducting the analyses separately for each subgroup. In the primary analysis, socioeconomic groups were classified using a standard Chilean SES classification provided by Kantar WorldPanel. Alternatively, we proxied for SES using the level of education of the household head, the occupation of the household head, and a composite indicator combining the occupation and the education of the household head and partner with asset ownership using a multivariate analysis (see S2 Text and S1 Fig) [32]. We also disaggregated household purchases by mean household BMI of adult members aged 18 years and over. We classified households into the following 3 groups: normal weight (<25 average BMI), overweight (≥25 average BMI and <30 average BMI), and obese (≥30 average BMI).

A final subgroup analysis looked at the changes among households with different levels of purchasing of beverages in the pretax period. We classified households into 3 categories based on their purchasing of high-tax items prior to the tax implementation.

## Results

Table 1 shows the descriptive statistics of products and households from January 2011 to December 2015.

**Table 1:** Descriptive information.

**A: Household Information**		
	**Proportion**	
**SES**		
High	35.8%	
Middle	30.2%	
Low	34.0%	
**Occupation**		
None	8.6%	
Unskilled	12.2%	
Skilled	64.8%	
Retired	14.4%	
**Education**		
None	26.8%	
Primary	21.4%	
Secondary	37.8%	
Tertiary	14.0%	
	**Mean**	**SD**
**Age**	48.32	14.76
**Family Size**	4.14	1.66
**BMI (Mean Adult)**	26.65	3.04
**Number of Households**	2,836	
**Number of Observations**	113,044	
**B: Monthly Purchase Information**		
	**Mean**	**SD**
**Volume (mL)**		
All soft drinks	7,349.5	6,839.5
High-tax soft drinks	3,574.8	4,407.7
Low-tax soft drinks	2,627.9	3,810.4
No-tax soft drinks	379.1	1,257.7
Sugar (g)	373.5	443.3
**C: Product Information**		
**Price (Chilean Peso) per 100 mL**		
All soft drinks	52.25	35.11
High-tax soft drinks	63.38	33.17
Low-tax soft drinks	39.88	34.3
No-tax soft drinks	41.4	23.41
Sugar (g contained per 100 mL)	6.02	5.28
**Number of Products (Observations)**		
All soft drinks	816 (37,699)	
High-tax soft drinks	386 (19,693)	
Low-tax soft drinks	384 (15,632)	
No-tax soft drinks	46 (2,374)	

Number of observations is based on outcomes over 60 months from January 2011 to December 2015. Volume (mL) of items purchased per capita per month. For sugar, the unit of measurement is g per 100 mL. 3. SES is that given by Kantar WorldPanel. Occupation, education, and age refer to the household head. Mean adult BMI is calculated for all household members older than 18 years. If a household did not participate in 2014, we prioritised the use of subsequent years (2015, 2013, 2012, 2011). The number of products is based on SKU; i.e., items with different barcodes (for example, items with the same ingredients but with different package sizes) are treated as distinct SKUs.

Abbreviations: BMI, body mass index; SES, socioeconomic status; SKU, stock-keeping unit.

The total number of products is 816, with 386 ‘high-tax’ products, 384 ‘low-tax’ products, and 46 ‘no-tax’ products. The composition of these tax categories by beverage category (i.e., soda, light soda, juice, or mineral water) is shown in S2 Table. The number of households in the sample is 2,836. S3 Table presents the descriptive statistics by socioeconomic group.

### Volume of soft drinks purchased

**Fig 1:**
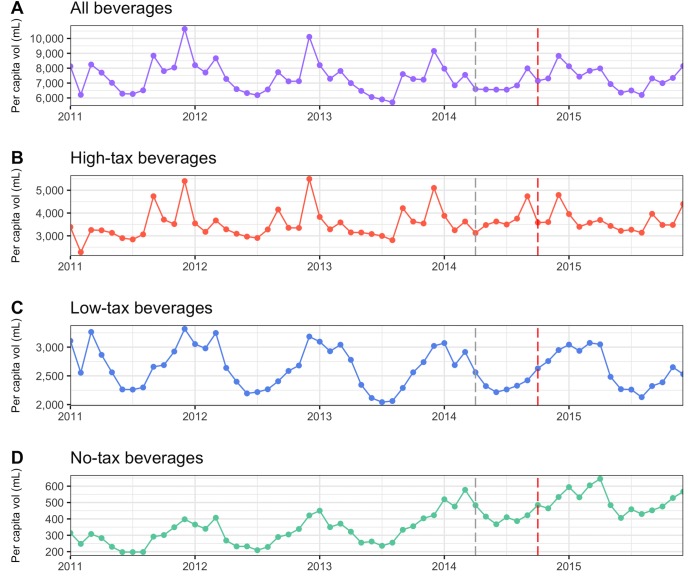
Volume (mL) of soft drink products purchased from January 2011 to December 2015. (A) All beverages, (B) high-tax products, (C) low-tax category, (D) no-tax category. Aggregated mean volume of soft drinks purchased from January 2011 to December 2015. The grey vertical dotted line in the figure refers to the announcement of the IABA tax policy in March 2014, whereas the red vertical dotted line is for the implementation of the policy in October 2014. Source: authors’ analysis of the Kantar WorldPanel data for urban households in Chile. IABA, Impuesto Adicional a las Bebidas Analcohólicas.

Fig 1 provides a descriptive, visual presentation of the aggregated mean volume of soft drinks purchased over time before and after the implementation of the tax policy (see S2 Fig for the figure for sugar).

For all soft drinks and for the relevant soft drink subcategories (except the no-tax soft drinks, which show a trend increase), it is hard to detect a clear overall time trend based on pure visual inspection alone. While the peaks in the data certainly become less pronounced over time, so have some of the troughs. It is equally difficult to discern an obvious pre- versus post-tax pattern in any of the categories.

Table 2 presents the results of the main regression analyses.

**Table 2:** Results from fixed-effects volume regression model (outcome in log mL).

	All	SES		
		Low	Middle	High
**All Soft Drink**	** **	** **	** **	** **
Point estimate	−0.060*	−0.043	−0.067	−0.057
Standard error	0.024	0.045	0.041	0.039
Proportionate change	−5.8%*	−4.2%	−6.5%	−5.5%
Pretax mean outcome (mL)	7,341.40	6,567.39	7,217.27	8,100.41
**High-Tax Soft Drink**				
Point estimate	−0.244***	−0.129	−0.179*	−0.376***
Standard error	0.044	0.073	0.076	0.078
Proportionate change	−21.6%***	−12.1%	−16.4%*	−31.3%***
Pretax mean outcome (mL)	3,544.84	3,524.01	3,715.24	3,427.45
**Low-Tax Soft Drink**				
Point estimate	0.030	−0.188	0.200	0.073
Standard error	0.062	0.114	0.119	0.094
Proportionate change	3.0%	−17.1%	22.1%	7.6%
Pretax mean outcome (mL)	2,627.91	2,059.60	2,422.93	3,275.52
**No-Tax Soft Drink**				
Point estimate	−0.105	−0.068	0.083	−0.270**
Standard error	0.054	0.088	0.102	0.092
Proportionate change	−10.0%	−6.6%	8.7%	−23.7%**
Pretax mean outcome (mL)	337.77	180.59	286.84	512.31
**Sugar**				
Point estimate	−0.164***	−0.093	−0.145**	−0.225***
Standard error	0.029	0.05	0.051	0.049
Proportionate change	−15.1%***	−8.9%	−13.5%**	−20.1%***
Pretax mean outcome (gr)	368.85	443.51	382.14	366.94
**Number of Households**	2,836	1,120	963	1,138
**Number of Observations**	113,044	36,443	34,010	42,591

The number of households in each SES group does not add to the total households due to movement between groups. Proportionate change = exp(point estimate) − 1.

*P < 0.05

**P < 0.01

***P < 0.001.

Abbreviation: SES, socioeconomic status.

Based on the principle of including the time polynomial that minimises the AIC, the main results included up to a fourth-order polynomial of time (see S4 Fig for a visual illustration of the fit of the model to the data). The results indicate a barely significant decrease in the volume of all soft drinks purchased but a highly significant decrease on the monthly purchased volume of high-tax soft drinks by 21.6% (i.e., the implied proportionate change, with the point estimate −0.244 and standard error 0.044). This corresponds to a reduction of 766 mL per person per month for an average household (the mean of pretax volume is 3,544.8 mL per person per month). The change in purchasing is also reflected in the significant 15.1% decrease in the amount of sugar purchased via soft drinks (point estimate −0.164; standard error 0.029). By contrast, we detected no significant change in either the volume of low-tax items or in the volume of no-tax items purchased. The alternative DiD regression approach largely confirms the results from our preferred fixed-effects approach (see S1 Table). In an assessment of the announcement of the tax, we found no significant changes in all, high-tax, and no-tax beverages and sugar purchasing, but we did see a decline in purchasing of low-tax soft drinks (see S4 Table).

For both high-tax soft drinks and the amount of sugar purchased, the overall change in volume appears to be driven by a decrease in the purchasing among the middle- and high-SES groups as well as in the high pretax purchasers, which saw a significant reduction in the volume of high-tax soft drinks of 16.4% (point estimate −0.179; standard error 0.076), 31.3% (point estimate −0.376; standard error 0.078), and 25.3% (point estimate 0.292; standard error 0.053), respectively (also see S5 Table). For the amount of sugar purchased, the magnitudes of the reductions were 13.5% (point estimate −0.145; standard error 0.051) for the middle-SES group, 20.1% (point estimate −0.225; standard error 0.049) for the high-SES group, and 23.6% (point estimate −0.212; standard error 0.038) for the high pretax purchasers, respectively (also see S5 Table). This pattern of results was robust to the different SES measures mentioned above (see S6 Table).

S7 Table presents the association between the tax implementation and the share of the expenditure of each tax group (e.g., high-tax soft drinks) out of the total soft drink expenditure. The results indicate that there was a shift in the composition of the soft drinks purchased post tax implementation, as the expenditure share of high-tax soft drinks decreased while that of low-tax items increased.

In S5 Table and S8 Table, we further disaggregated the main analysis by splitting the sample by risk factor groups, according to either the mean BMI of adults in the households or the level of per capita purchasing of high-tax soft drinks before the tax. The reduction in purchasing of high-tax items is statistically significant in all BMI groups, and there was no systematic patterning in the magnitude of the effect across groups. For groups categorised according to their pretax purchasing volume of high-tax soft drinks, there was a significant reduction of similar size in the middle and highest groups and no statistically significant impact among those in the lowest group.

Further results reported in S9 Table and S10 Table explored the sensitivity of the point estimates to different choices of functional form of the time trend. All the models with the full sample indicated a reduction in the volume of high-tax soft drinks and the amount of sugar purchased, but the reduction was only statistically significant using higher order (fourth and fifth) polynomials, as well as a linear time trend. By SES subgroup, for the high-tax soft drinks, all models showed a significant volume decrease that varied from −12.8% to −31.3% in the high-SES subgroup and from −6.9% to −21.2% in the high pretax volume purchasers. In the low pretax volume purchasers, all the model specifications indicated a nonsignificant change for the high-tax soft drinks and the amount of sugar purchased. For the other subgroups, in both the volume of high-tax soft drinks purchased and the volume of sugar purchased, a significant decrease was found in some models, while no significant change was observed in other model specifications. For low-tax soft drinks, the full sample models indicated a nonsignificant change or a significant increase of up to 14.6% in the purchased volume. In the middle-SES, the low-BMI, and the low pretax volume subgroups, the results varied from a nonsignificant change to a significant increase of up to 25.2%, 21.1%, and 18.1%, respectively. For the other subgroups, all the models indicated nonsignificant changes.

### Paid price

S3 Fig presents the descriptive picture of the prices (per 100 mL) of the relevant soft drink categories that were paid by consumers, rather than those that were posted in stores. Overall, there was an upward trend in prices for all tax categories. For the high-tax item category, we observed an increase precisely after the announcement of the policy (end of March 2014), which was also seen in the all-soft drink category. After the implementation of the policy, however, the lines appear to revert back to the longer-term trend.

Table 3 provides the regression results for price, distinguishing between a potential ‘implementation effect’ and an ‘announcement effect’.

**Table 3:** Price regression model (log price/mL) with date of implementation and date of announcement.

	All	High-Tax	Low-Tax	No-Tax
Point estimate (implement)	−0.010	−0.008	−0.017*	0.017
Standard error	0.01	0.01	0.01	0.02
Proportionate change (implement)	−1.0%	−0.8%	−1.7%*	1.7%
Point estimate (announcement)	0.016**	0.019*	0.013	0.001
Standard error	0.01	0.01	0.01	0.02
Proportionate change (announcement)	1.6%**	1.9%*	1.3%	0.1%
Mean outcome (log price/mL)	0.388	0.507	0.278	0.339
**Number of Products**	816	386	384	46
**Number of Observations**	37,699	19,693	15,632	2,374

Implement refers to September 2014 and Announcement refers to March 2014. Proportionate change = exp(point estimate) − 1.

*P < 0.05

**P < 0.01

***P < 0.001.

The results show that prices of all soft drinks and of the high-tax soft drinks increased after the announcement, though not after implementation. The magnitude of the price change was a 1.6% increase for all soft drinks (point estimate 0.016; standard error 0.01) and a 1.9% (point estimate 0.019; standard error 0.01) increase for high-tax items. In addition, the price of low-tax items declined by 1.7% (point estimate 0.017; standard error 0.01) upon tax implementation.

S11 Table provides the results of the price regressions by SES. As with our pooled results, the results by SES show that there were few changes in the price of each type of item. Notably, whereas before we saw a modest decrease in the price of low-tax items after the implementation, this effect now disappears. There was a 2% to 3% decline, however, in the price of high-tax items for the middle- and low-SES groups.

### Shopping pattern

Going beyond the analysis of the volume of soft drinks purchased, we also sought to understand some of the potential mechanisms behind the observed changes. In particular, we examined changes in price and in certain shopping patterns. In S12 Table, we examined the association between the tax implementation and the number of days per month involving purchases of any soft drinks, i.e., the frequency of shopping trips. The frequency of shopping trips that included high-tax soft drinks decreased by 7.8% (point estimate −0.081; standard error 0.011), and these changes are visible in all SES groups, though they are more significant and larger in the middle- and high-SES groups. The change in the frequency of shopping trips involving low-tax soft drinks was only significant for the high-SES group, with a 5.0% reduction (point estimate −0.051; standard error 0.016).

Furthermore, we explored whether one way for consumers to ‘cope’ with the tax increase was to revert increasingly to the purchase of price-promoted items. Such behaviour seemed to unfold both in relation to high-tax and low-tax soft drinks. In S13 Table, we observe a less than 1% increase in the use of price promotions, which appears to be driven by the high-SES group for high-tax items.

In S14 Table, the main analysis was separately conducted by product type, with categories including ready-to-drink, concentrated, carbonated, and noncarbonated beverages. The type of retailer—major chain store or local, independent store—was also examined. The results show that, first, the association between purchasing and tax implementation was pronounced for ready-to-drink items, and there was only a small, nonsignificant change for concentrated items. Second, the magnitude of the reduction in the volume of all or high-tax soft drinks purchased was larger for carbonated drinks than it was for noncarbonated items. Finally, the volume of items purchased from major chain stores declined more after the tax policy than those from local or independent stores.

## Discussion

This study assessed the short-term (12 months after the implementation) changes in household-level purchasing of soft drinks after the Chilean SSB tax, controlling for regional economic conditions, regional temperature, and long-term seasonal fluctuations, as well as households’ time-invariant characteristics. Our main estimates suggest a significant, sizeable reduction in the volume of high-tax soft drinks purchased (21.7%), which was also reflected in a decrease in the amount of purchased added sugar from soft drinks (15.1%). In contrast, we did not find any significant increase in the volume of low-tax items purchased.

The specific tax policy implemented by Chile is unique worldwide in that it (1) combines increases in the tax for sugary soft drinks with decreases in the tax for less sugary ones and (2) uses a specific threshold of added sugar concentration (6.25 g per 100 mL) to increase taxes by 5% for those beverages above, and to decrease taxes by 3% for those below, the threshold. By contrast, the recent SSB tax policies in Mexico and in California proportionately linked the tax to the overall volume of the beverage with added sugar. The magnitude of the tax incentive also appears more modest than the recommended 20% tax that some organisations and researchers have called for [33,34]. The finding that there appears to have been a short-term change in purchasing suggests that, first, even more modest tax incentives have the potential to reduce purchasing of SSBs and, second, that there may be more than one way in which an SSB tax can be implemented with some success.

The fact that we found no consistent associations between the tax implementation and the purchased volume of low-tax soft drinks may hint at the presence of asymmetries in the price response between tax and subsidies, in contrast to the traditional assumption of symmetric price elasticities. Prospect theory, developed by Kahneman and Tversky [35], may help explain the phenomenon because it argues that consumers respond more strongly to a price increase than to a price decrease, starting from the consumers’ internal reference price, against which they assess the actual price of the beverage [36].

The reduction in purchasing of soft drinks shown in Table 2 is fairly widespread across SES groups. Notably, for the volume of high-tax items purchased, the magnitude of reduction was larger for the high-SES group than for the middle-SES group and statistically insignificant for the low-SES group. This may be surprising to those who expect the response to be greatest among those with the tightest budget constraints, i.e., those among the lower-SES group. On the other hand, theoretical considerations also predict that higher- and middle-SES groups could be better placed either to make cost-minimising purchasing decisions and/or to better absorb and act upon the information conveyed by the tax change (i.e., that certain beverages are deemed unhealthy) [37]. While many price elasticity studies, including one in Chile, show that individuals or households at lower incomes are more elastic than those at higher incomes [38,39], other studies—especially some that use randomised designs—reject the commonly expected pattern in favour of a more even responsiveness across socioeconomic groups [33,40,41].

Disaggregating the analyses by obesity status of the household, we found that all groups responded to the tax by cutting down their purchasing of high-tax soft drinks. When disaggregated by volume of high-tax soft drinks purchased in the pretax periods, all but the lowest category of high-tax soft drink purchasers significantly reduced the volume of high-tax items purchased. As is shown in Table 2, the baseline level of high-tax soft drink volume purchased was similar between the high- and low-SES groups, and within the group of high-tax soft drink purchasers, there was considerable representation of households from all SES categories. Disaggregating the results further by splitting the low, medium, and high volume pretax purchasers of soft drinks into low-, medium-, and high-SES groups, it turned out that what may be driving the responsiveness within the high volume purchasers was the responsiveness of the high-SES groups (see S15 Table). This is consistent with the greater overall change after the tax among the high-SES group. However, again, there is some degree of sensitivity of these results to the assumptions about the functional form of the time trend.

For the first time that we are aware of in a study evaluating SSB taxation, this work has examined more facets of the behavioural response pattern. First, we showed that households decreased the frequency of shopping trips that included high-tax soft drinks and, perhaps as a way of coping with the tax, households modestly increased the use of price promotions.

We found no major change in the price of soft drinks that were paid by the households. Several factors may explain this finding. First, as emphasised before, the data only capture the price of those items that are actually purchased. Consumers may have accepted a given price only when it was at or below a level prior to the tax implementation, leading to a smaller amount of high-tax soft drinks purchased without showing a significant increase in the prices paid. This does remain compatible with the possibility that the prices posted in the stores increased, which we could not examine in detail with the data at hand. Secondly, because the SSB tax is an ad valorem tax with 2 different rates, it is possible that manufacturers reacted strategically to partially or fully absorb the additional tax burden and did not pass-through the tax to the final price—at least for the popular items we covered in this analysis—or they could also have offset the price increase in the high-tax soft drinks via a smaller reduction in the price of the low-tax items. Thirdly, there could be a mechanism at work producing behavioural change that works independently of any price effect (e.g., via signalling of certain products as ‘unhealthy’). Finally, the SSB tax was only a minor part of a major tax reform, thus limiting its public visibility, and those who have better access to information—such as those in higher SES groups—may have been more likely to be aware of the SSB tax and react to it by reducing purchases upon implementation. In S3 Text, S5 Fig, and S6 Fig, we present a time series of internet searches for soft drink–and tax policy–related themes as a measure of public interest and information-seeking behaviour, as retrieved from Google Trends. A modestly higher frequency of internet searches for the related keywords was observed immediately after the policy announcement and implementation. It may therefore be possible—though difficult to test formally—that access to media and information sources, which tends to be greater among higher-SES groups, might partially explain the differences in purchasing of SSBs across SES groups after the tax implementation.

### Strengths and limitations

A major strength of this evaluation is the quality of the data used, which are arguably the best available for a short-term SSB purchasing evaluation of this tax policy. The data contain detailed information of purchases of soft drinks at the household level as well as a range of important household characteristics. Considerable human resources and efforts have been invested into the collection of the data, with interviewers regularly visiting households that were part of the panel. The methods used to analyse this data are also appropriate statistical techniques widely used in the quasi-experimental policy evaluation literature, and they are comparable to approaches employed in previous, similar policy evaluations [14].

In interpreting the results, several limitations need to be considered. First, because this is an evaluation without a randomised design, it suffers from the lack of a proper control group in the strict sense. We cannot exclude the possibility of not capturing the influence of time-varying unobservable factors. For example, we could not observe whether and how marketing strategies (such as advertisement) might have changed after the announcement and implementation of the tax policy. Also, a new tax on alcoholic beverages was implemented at the same time, which could be problematic for our SSB tax evaluation if there was substitution between soft drinks and alcohol items. Because the tax reform also introduced several changes in other taxes (e.g., income taxation) that might have had an impact on the economy, this could be another source of endogeneity that cannot be entirely ruled out in the present analysis.

Also, when interpreting the impacts found in this study, regression to the mean (RTM) could be a concern [42]. RTM would occur if households whose pretax purchasing of soft drinks became particularly high due to unobserved shocks or measurement errors. Those households would show decreased purchasing even in the absence of the policy implementation, and if this was the case, then the estimates could have been overstated. In S7 Fig, S8 Fig, S9 Fig and S10 Fig, we checked whether the trends (with standard deviations) of log-purchasing of soft drinks appeared stable—separately by SES and the volume of pretax purchasing group—in order to assess whether RTM might play a role. The figures show that the pretax purchasing was, overall, quite stable in terms of both level and deviation over the pretax period for all groups, implying that RTM is unlikely to explain the results.

Secondly, the nutritional information that we used to classify the beverages into the relevant tax categories was not originally part of the Kantar WorldPanel data but was collected by the research team using national databases and captured only the items among the top 90% of sales in the data. This means that our results predominantly represent consumers’ purchasing of major items in the market, and not necessarily their purchasing all items.

Thirdly, the data captured household purchasing rather than what would ultimately affect health outcomes, i.e., actual consumption. Moreover, given that the data are at household level, we do not have information about how foods and beverages were allocated within the household.

Fourthly, the data only cover households living in urban areas (North, Valparaiso Region, Center South, Bio-Bio Region, and South). Whether or not purchasing of SSBs in rural areas decreased after the tax implementation remains unknown; however, it is worth pointing out that 90% of Chile’s population in 2016 was living in urban areas [43].

Finally, in the data, it is not possible to identify the reasons why a given household dropped out of the survey. For example, if there were households dropping out due to health problems, they might have had chronic conditions as a result of a poor diet—including excess consumption of sugary drinks—in which case our data might underrepresent those households [44]. Although it is hard to predict the direction and magnitude of bias that may result from omitting those households, this is certainly a limitation of the study. Because the prevalence of high-volume SSB drinkers can be expected to be higher in the lower-SES group, health-related attrition could potentially explain the heterogeneous findings across SES groups.

### Implications for future research

While we applied a standard analytical approach to the best available data of SSB purchasing in Chile, there is scope for further data collection and analyses. The data comprise less than 3,000 households that do not necessarily buy soft drinks regularly. Given the relatively limited variations of purchasing, the within-household estimates of the changes in soft drink purchasing for the full sample and certain subgroups could be subject to a degree of uncertainty (and larger standard errors), depending on the functional forms assumed for the time trend in the regression models. Nevertheless, the differences observed are in terms of the statistical significance and the magnitude of the point estimates, but typically not in the direction of the changes. Further data collection (which has been beyond the feasibility of the present research) would likely reduce the uncertainty and increase the precision of the estimates.

Any changes in soft drink purchasing resulting from an SSB tax would be expected to entail interactions, possibly substitutions between soft drinks and food items. In this study, we were not able to examine the extent to which the tax policy was associated with the purchased amount of overall sugar contained in all foods and beverages. For example, it could be the case that those who reduced sugar from soft drinks did increase their sugar intake from confectionaries. Future research should explicitly consider nutrient information of food items as well as of soft drinks and examine the role of the SSB tax in overall sugar intake from beverages and foods. SSB intake accounts for about one quarter of the total sugar consumed by the Chilean population [45], implying that a more broad-based sugar tax (also covering added sugar in solid foods), or even a tax on energy-dense and ultra-processed foods more generally, may be more effective in improving diet-related health outcomes. In this context, modelling the anticipated health impacts of different tax strategies in Chile would be a relevant future direction for research.

Finally, the use of fiscal incentives to combat obesity and related NCDs has now become a promising policy tool in the eyes of many countries and of the World Health Organization, even though only a few jurisdictions have hitherto implemented such policies [34]. To date, the evaluative literature of SSB tax policies, including our present study, focusses on purchasing with relatively short-term follow-up periods after the tax policies. Future evaluations should include the impact of the policies on consumption and health outcomes. Currently, the evidence on health outcomes (e.g., BMI) is typically based on epidemiological models that rely on many assumptions and embody a substantial degree of uncertainty. Longer-term assessments are needed, not least because the behavioural changes may either shrink or grow in the long run [46]. The apparent association of the Chilean SSB tax with a reduction of purchases of SSBs—and, in particular, the amount of added sugar in the short run—may nevertheless be considered a promising result from a policy perspective.

### Implications for policy

Previous studies focussed on evaluating the impact of the excise tax on purchases and found some significant short-term reductions [14,17]. Our study evaluated an ad valorem tax and also found some signs of a significant reduction in purchases. This might imply that the nature of the tax—specific rate tax or ad valorem tax—would not critically affect the effectiveness of the tax policy. However, in judging the full ‘success’ of the tax policy and in considering future potential improvements, it needs to be borne in mind what the ultimate purpose of a corrective tax should be, from an economic perspective, to help increase the price of SSBs so as to align their full ‘social costs’—which include costs to society, the economy, health, and the environment—with the costs that are perceived by the individual. Such social costs could entail the costs of future collectively funded extra health care costs to treat diet-related diseases or the future health problems not anticipated by the individual consumer. With this in mind, it is hard to argue against the notion that the higher the added sugar content in a given beverage, the higher the social costs associated with the consumption of this beverage will be, and therefore the higher the taxed amount should be. In light of the current threshold nature of the Chilean SSB tax, those who consume SSBs with particularly large amounts of added sugar (i.e., well above 6.25 g per 100 mL) are not discouraged more than those who consume SSBs just above the threshold. Similarly, those who consume SSBs just below the threshold are encouraged to increase consumption. Although we did find that the amount of added sugar from soft drinks purchased decreased overall, it is still far from clear that this tax design is best suited to maximise population health and social welfare.

The present study is the first to evaluate a reduction of the tax on ‘low-tax’ soft drinks implemented in the real world. The results imply that consumers may not significantly respond to a modest tax subsidy (by 3%) for less sugary soft drinks but may respond to a tax of similar size (by 5%). The tax gap of 8% between high- and low-tax items did not appear to induce considerable substitutions between items in respective categories. A recent WHO report states that SSB prices would need to be raised by 20% or more in order to achieve meaningful health effects [34], a suggestion that may have been informed by previous studies examining the impact of a 20% tax on SSBs [33,47]. In the Chilean case, however, the rate was far below this level.

The robust subgroup results for the high-SES groups suggest that the policy may have been partially effective, though not necessarily likely to reduce socioeconomic inequalities in diet-related health given that higher-SES groups appeared to have cut down on sugary drink purchasing while the lower-SES groups have not. This could also imply that the financial burden of the tax is predominantly carried by the latter, increasing the chances that the policy will have regressive financial impacts. Ultimately, policy makers will need to decide whether the expected gains in diet-related health for the higher-SES groups would compensate for potentially increased inequities in diet-related population health.

## Supporting information

S1 Fig Correspondence between MCA-SES index and Kantar-SES index. MCA, multiple correspondence analysis; SES, socioeconomic status.
(DOCX)

S2 Fig Volume of sugar purchased from soft drinks over time. (DOCX)

S3 Fig Paid price of soft drinks over time. (DOCX)

S4 Fig A visual illustration of the fit of the main model: Raw data (log volume of SDs purchased) versus predicted values. SD, sugary drink.
(DOCX)

S5 Fig Internet search of ‘bebida’ and related terms. (DOCX)

S6 Fig Internet search of composite terms. (DOCX)

S7 Fig Trend of log-volume of all soft drinks purchased, by pretax purchasing volume of high-tax items. (DOCX)

S8 Fig Trend of log-volume of high-tax soft drinks purchased, by pretax purchasing volume of high-tax items. (DOCX)

S9 Fig Trend of log-volume of all soft drinks purchased, by socioeconomic group. (DOCX)

S10 Fig Trend of log-volume of high-tax soft drinks purchased, by socioeconomic group. (DOCX)

S1 Table Quasi DiD results for volume of soft drinks purchased. DiD, difference-in-difference.
(DOCX)

S2 Table Composition of tax categories by 4 beverage categories. (DOCX)

S3 Table Descriptive statistics by SES group. SES, socioeconomic status.
(DOCX)

S4 Table Changes in volume of soft drinks purchased after the policy announcement. (DOCX)

S5 Table Regression analysis using alternate measures of pretax expenditure of soft drinks. (DOCX)

S6 Table Regression analysis using alternate measures of SES group. SES, socioeconomic status.
(DOCX)

S7 Table Regression analysis for the share of expenditure of each tax group in total expenditure on soft drinks. (DOCX)

S8 Table Regression analysis using alternate measures of BMI group. BMI, body mass index.
(DOCX)

S9 Table Sensitivity checks for the polynomial function using AIC: Regression model for volume of soft drinks. AIC, Akaike Information Criterion.
(DOCX)

S10 Table Sensitivity checks for the polynomial function using AIC: Regression model for price of soft drinks. AIC, Akaike Information Criterion.
(DOCX)

S11 Table Regression analysis for price paid by SES. SES, socioeconomic status.
(DOCX)

S12 Table Regression analysis for frequency of shopping of soft drinks per month. (DOCX)

S13 Table Regression analysis for use of price promotions. (DOCX)

S14 Table Volume regression results by product type ([A] ready-to-drink and concentrated; and [B] carbonated and noncarbonated) and by type of retailer ([C] major store chain and local or private store).
(DOCX)

S15 Table Regression analysis for volume of soft drinks purchased by further disaggregated subgroup. (DOCX)

S1 Text A quasi-DiD approach. DiD, difference-in-difference.
(DOCX)

S2 Text Alternative SES measures. SES, socioeconomic status.
(DOCX)

S3 Text Trend of keyword search of the IABA tax policy on the internet. IABA, Impuesto Adicional a las Bebidas Analcohólicas.
(DOCX)

S1 STROBE STROBE statement: Checklist of items that should be included in reports of observational studies. (DOCX)

## Abbreviations

AIC: Akaike Information Criterion
BMI: body mass index
DiD: difference-in-difference
IABA: Impuesto Adicional a las Bebidas Analcohólicas
NCD: noncommunicable disease
RTM: regression to the mean
SES: socioeconomic status
SKU: stock-keeping unit
SSB: sugar-sweetened beverage
VAT: value-added tax
