# Supplementary material for: Evaluating the 2014 sugar-sweetened beverage tax in Chile: An observational study in urban areas
Source: PLoS Med. 2018 Jul 3;15(7):e1002596. doi: 10.1371/journal.pmed.1002596 (PMC6029775; doi:10.1371/journal.pmed.1002596)
Supplement: S1 Fig — MCA, multiple correspondence analysis; SES, socioeconomic status. (DOCX) [file pmed.1002596.s001.docx]

**S1 Fig**

**Correspondence between MCA-SES index and Kantar-SES index**
