# Supplementary material for: Evaluating the 2014 sugar-sweetened beverage tax in Chile: An observational study in urban areas
Source: PLoS Med. 2018 Jul 3;15(7):e1002596. doi: 10.1371/journal.pmed.1002596 (PMC6029775; doi:10.1371/journal.pmed.1002596)
Supplement: S2 Fig — (DOCX) [file pmed.1002596.s002.docx]

**S2 Fig**

**Volume of sugar purchased from soft drinks over time**

**
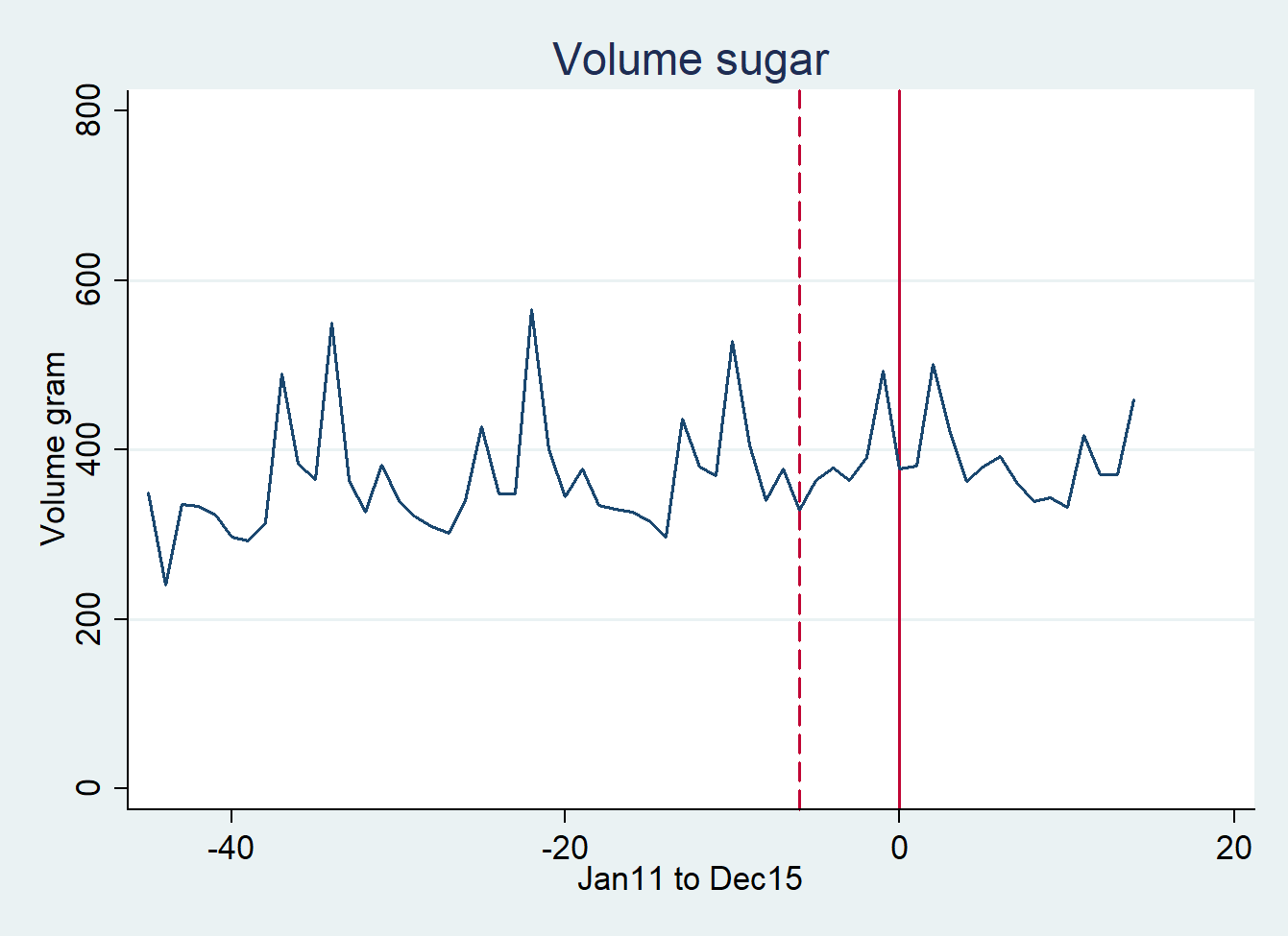
**

Note: The dashed and solid red line show the announcement and the implementation of the tax policy, respectively.
