# Supplementary material for: Evaluating the 2014 sugar-sweetened beverage tax in Chile: An observational study in urban areas
Source: PLoS Med. 2018 Jul 3;15(7):e1002596. doi: 10.1371/journal.pmed.1002596 (PMC6029775; doi:10.1371/journal.pmed.1002596)
Supplement: S4 Fig — SD, sugary drink. (DOCX) [file pmed.1002596.s004.docx]

**S4 Fig**

**A visual illustration of the fit of the main model - raw data (log volume of SDs purchased) vs. predicted values**


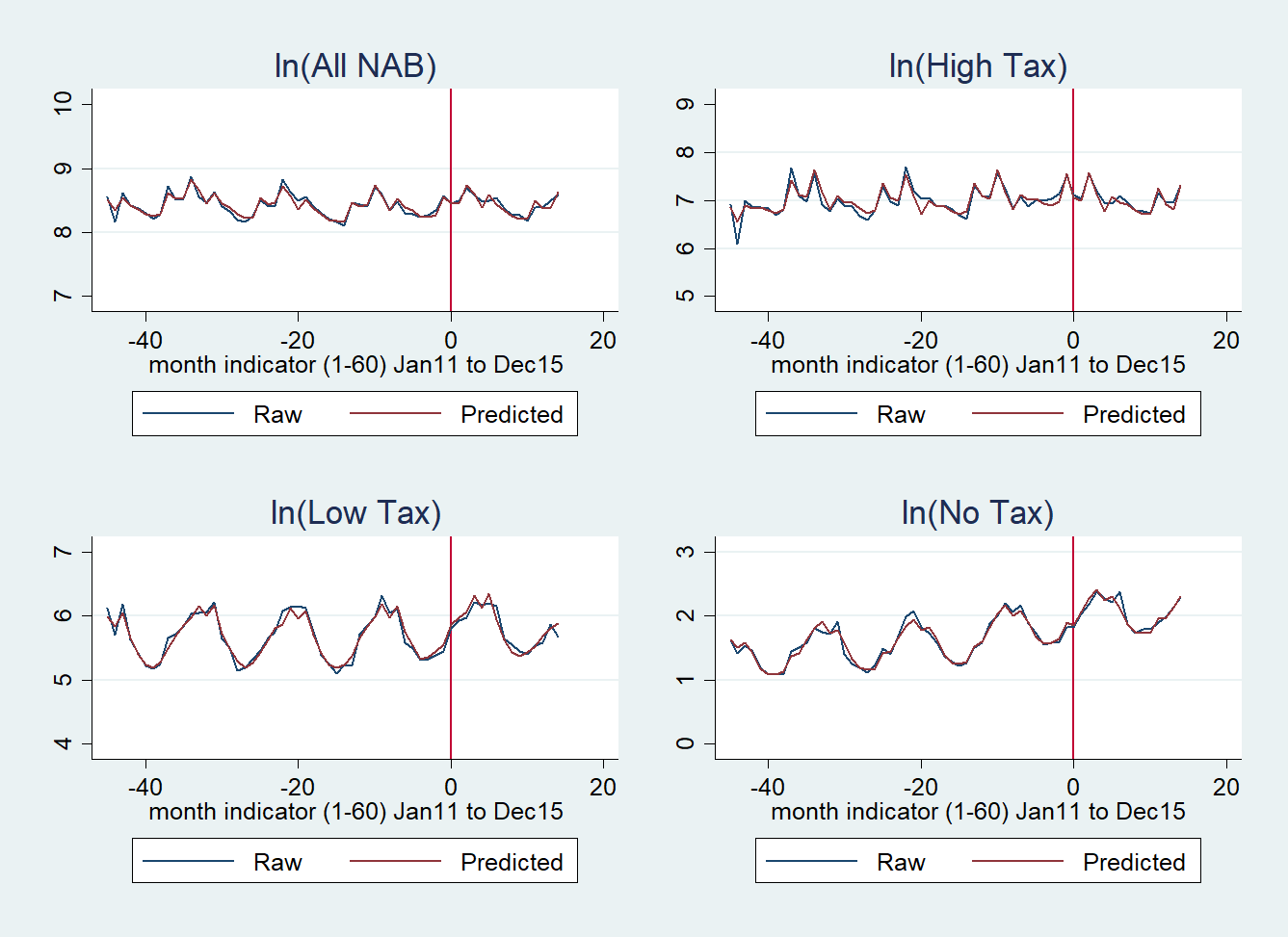


Note: Vertical axis is logged per capita monthly volumes (mL). Black line – actual logged purchases from Kantar WorldPanel. Red line – predicted values from regression model.
