# Supplementary material for: Evaluating the 2014 sugar-sweetened beverage tax in Chile: An observational study in urban areas
Source: PLoS Med. 2018 Jul 3;15(7):e1002596. doi: 10.1371/journal.pmed.1002596 (PMC6029775; doi:10.1371/journal.pmed.1002596)
Supplement: S5 Fig — (DOCX) [file pmed.1002596.s005.docx]

**S5 Fig**

**Internet search of “bebida” and related terms**


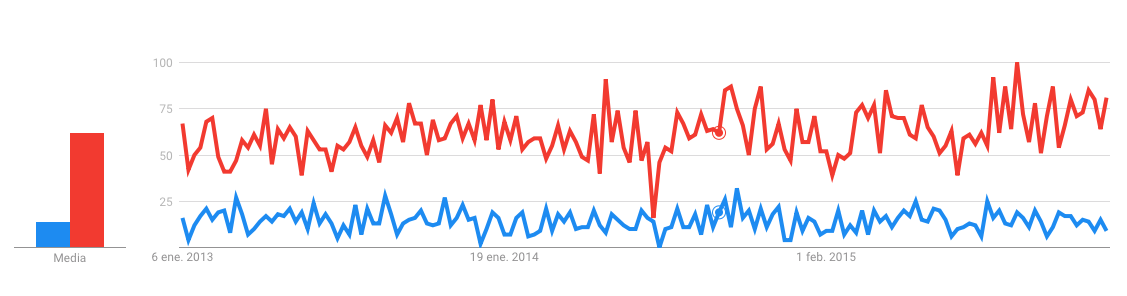


Source: https://trends.google.es/trends/explore?date=2013-01-01%202015-12-31&geo=CL&q=%2Fm%2F06qrr,bebida
