# Supplementary material for: Evaluating the 2014 sugar-sweetened beverage tax in Chile: An observational study in urban areas
Source: PLoS Med. 2018 Jul 3;15(7):e1002596. doi: 10.1371/journal.pmed.1002596 (PMC6029775; doi:10.1371/journal.pmed.1002596)

**S7 Fig**

**Trend of log-volume of all soft drinks purchased, by pre-tax purchasing volume of high-tax items**


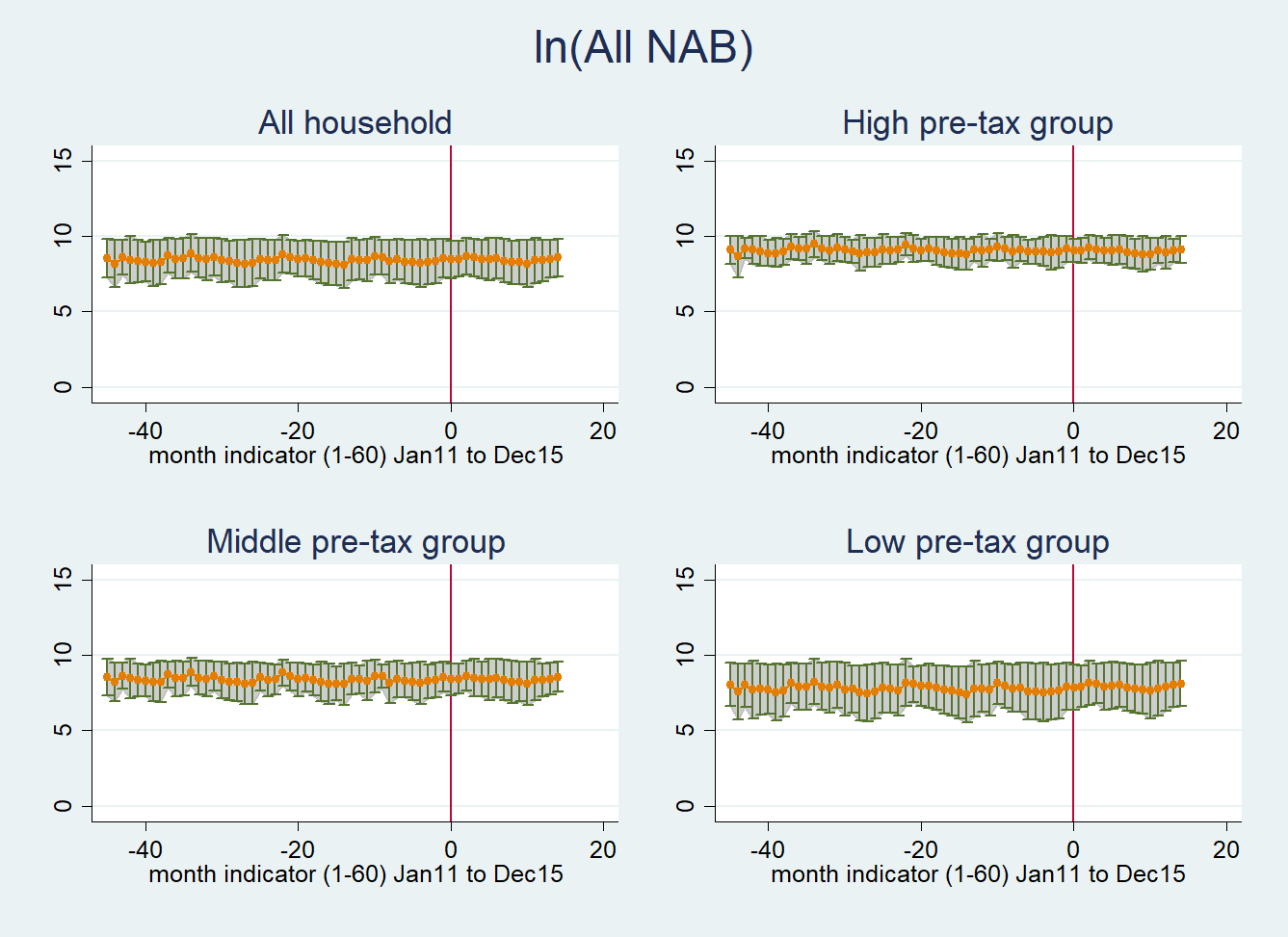

Supplement: S7 Fig — (DOCX) [file pmed.1002596.s007.docx]
