# Supplementary figures and images for: Evaluating the 2014 sugar-sweetened beverage tax in Chile: An observational study in urban areas
Source: PLoS Med. 2018 Jul 3;15(7):e1002596. doi: 10.1371/journal.pmed.1002596 (PMC6029775; doi:10.1371/journal.pmed.1002596)

**S9 Fig**

**Trend of log-volume of all soft drinks purchased, by socioeconomic group**


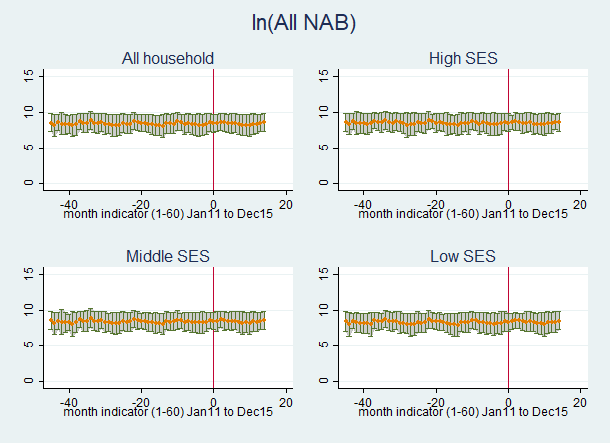

Supplement: S9 Fig — (DOCX) [file pmed.1002596.s009.docx]

**S10 Fig**

**Trend of log-volume of high-tax soft drinks purchased, by socioeconomic group**


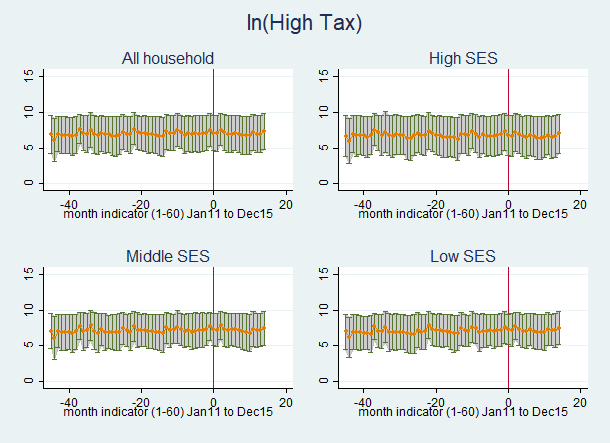

Supplement: S10 Fig — (DOCX) [file pmed.1002596.s010.docx]
