# Supplementary material for: Evaluating the 2014 sugar-sweetened beverage tax in Chile: An observational study in urban areas
Source: PLoS Med. 2018 Jul 3;15(7):e1002596. doi: 10.1371/journal.pmed.1002596 (PMC6029775; doi:10.1371/journal.pmed.1002596)
Supplement: S1 Table — DiD, difference-in-difference. (DOCX) [file pmed.1002596.s011.docx]

**S1 Table**

**Quasi difference-in-differences results for volume of soft drinks purchased**

|  | **All** |  | **SES** | | |
| --- | --- | --- | --- | --- | --- |
| **All Soft Drink** |  |  | **Low** | **Middle** | **High** |
| Point Estimate | -0.033 |  | 0.000 | -0.025 | -0.056 |
| Standard Error | 0.025 |  | 0.047 | 0.041 | 0.039 |
|  |  |  |  |  |  |
| **High Tax Soft Drink** |  |  |  |  |  |
| Point Estimate | -0.242 *** |  | -0.07 | -0.135 | -0.428*** |
| Standard Error | 0.044 |  | 0.074 | 0.073 | 0.077 |
|  |  |  |  |  |  |
| **Low Tax Soft Drink** |  |  |  |  |  |
| Point Estimate | -0.102 |  | -0.236* | -0.019 | -0.049 |
| Standard Error | 0.062 |  | 0.113 | 0.123 | 0.094 |
|  |  |  |  |  |  |
| **No Tax Soft Drink** |  |  |  |  |  |
| Point Estimate | -0.136* |  | -0.044 | 0.021 | -0.307*** |
| Standard Error | 0.055 |  | 0.090 | 0.104 | 0.093 |
|  |  |  |  |  |  |
| **Sugar** |  |  |  |  |  |
| Point Estimate | -0.149 *** |  | -0.051 | -0.110* | -0.237*** |
| Standard Error | 0.029 |  | 0.051 | 0.050 | 0.048 |
|  |  |  |  |  |  |
| **Number Households** | 2836 |  | 1120 | 963 | 1138 |
| **Number Observations** | 45714 |  | 14514 | 14085 | 17115 |

Note: * p<0.05, **p<0.01, *** p<0.001
