# Supplementary material for: Evaluating the 2014 sugar-sweetened beverage tax in Chile: An observational study in urban areas
Source: PLoS Med. 2018 Jul 3;15(7):e1002596. doi: 10.1371/journal.pmed.1002596 (PMC6029775; doi:10.1371/journal.pmed.1002596)
Supplement: S2 Table — (DOCX) [file pmed.1002596.s012.docx]

**S2 Table**

**Composition of tax categories by four beverage categories**

|  | **No Tax** | **Low Tax** | **High Tax** |
| --- | --- | --- | --- |
| **Mineral Water** | 100% | 10% | 0% |
| **Juice** | 0% | 71% | 26% |
| **Light Soda** | 0% | 19% | 0% |
| **Soda** | 0% | 0% | 74% |
| **Total** | 100% | 100% | 100% |

Note: The numbers in the table show the proportion of # of products of a given beverage category in the total # of items in the tax category group. For example, In no-tax category, mineral water accounts for 100% of the category.
