# Supplementary material for: Evaluating the 2014 sugar-sweetened beverage tax in Chile: An observational study in urban areas
Source: PLoS Med. 2018 Jul 3;15(7):e1002596. doi: 10.1371/journal.pmed.1002596 (PMC6029775; doi:10.1371/journal.pmed.1002596)
Supplement: S3 Table — SES, socioeconomic status. (DOCX) [file pmed.1002596.s013.docx]

**S3 Table**

**Descriptive statistics by socioeconomic status group**

|  | **All** | | **High SES** | | **Middle SES** | | **Low SES** | |
| --- | --- | --- | --- | --- | --- | --- | --- | --- |
| ***A. Household Information*** |  |  |  |  |  |  |  |  |
|  | **Proportion** | | **Proportion** | | **Proportion** | | **Proportion** | |
| **Occupation** |  |  |  |  |  |  |  |  |
| None | 8.6% | | 8.9% | | 7.4% | | 9.3% | |
| Unskilled | 12.2% | | 2.5% | | 8.9% | | 25.4% | |
| Skilled | 64.8% | | 71.6% | | 70.2% | | 53.0% | |
| Retired | 14.4% | | 17.1% | | 13.6% | | 12.3% | |
| **Education** |  |  |  |  |  |  |  |  |
| None | 26.8% | | 7.1% | | 29.5% | | 45.3% | |
| Primary | 21.4% | | 13.9% | | 26.2% | | 25.1% | |
| Secondary | 37.8% | | 46.5% | | 38.1% | | 28.4% | |
| Tertiary | 14.0% | | 32.6% | | 6.2% | | 1.2% | |
|  | **Mean** | **Std. Dev.** | **Mean** | **Std. Dev.** | **Mean** | **Std. Dev.** | **Mean** | **Std. Dev.** |
| Age | 48.32 | 14.76 | 51.16 | 14.39 | 48.76 | 14.40 | 44.96 | 14.88 |
| Family Size | 4.14 | 1.66 | 3.81 | 1.48 | 4.20 | 1.70 | 4.43 | 1.74 |
| BMI (mean adult) | 26.65 | 3.04 | 25.87 | 2.70 | 26.94 | 3.01 | 27.23 | 3.22 |
| Number of Households | 2,836 | | 1,016 | | 855 | | 965 | |
| ***B. Purchase Information*** |  |  |  |  |  |  |  |  |
| **Volume (ml)** |  |  |  |  |  |  |  |  |
| All Soft Drinks | 7349.5 | 6839.5 | 8048.9 | 7248.9 | 7265.1 | 6719.7 | 6610.9 | 6358.7 |
| High Tax Soft Drinks | 3574.8 | 4407.7 | 3398.2 | 4153.8 | 3777.9 | 4494.2 | 3591.7 | 4601.8 |
| Low Tax Soft Drinks | 2627.9 | 3810.4 | 3273.3 | 4467.5 | 2426.9 | 3562.9 | 2061.4 | 3003.6 |
| No Tax Soft Drinks | 379.1 | 1257.7 | 559.3 | 1651.5 | 334.3 | 1032.0 | 210.2 | 810.3 |
| Sugar (g) | 373.5 | 443.3 | 366.8 | 431.5 | 389.7 | 446.5 | 366.1 | 453.5 |

Notes: 1. Number of observations is based on outcomes over 60 months from January 2011 to December 2015. 2. Volume (ml) of items purchased per capita per month. For sugar the unit of measurement is mg per 100ml. 3. Socioeconomic Status is that given by Kantar WorldPanel. Occupation, Education and Age refer to the household head. Mean adult BMI is calculated for all household members older than 18 years. If a household did not participate in 2014, we prioritised use of subsequent years (2015, 2013, 2012, 2011). 4. The number of products is based on stock-keeping-unit (SKU), i.e. items with different barcodes (for example, items with the same ingredients but with different package sizes) are treated as distinct SKU.
