# Supplementary material for: Evaluating the 2014 sugar-sweetened beverage tax in Chile: An observational study in urban areas
Source: PLoS Med. 2018 Jul 3;15(7):e1002596. doi: 10.1371/journal.pmed.1002596 (PMC6029775; doi:10.1371/journal.pmed.1002596)
Supplement: S4 Table — (DOCX) [file pmed.1002596.s014.docx]

**S4 Table**

**Changes in volume of soft drinks purchased after the policy announcement**

| **All Soft Drink** |  |  |  |  |  |
| --- | --- | --- | --- | --- | --- |
|  | **All** |  | **SES** | | |
|  |  |  | **Low** | **Middle** | **High** |
| Point Estimate | 0.012 |  | -0.017 | 0.046 | 0.013 |
| Standard Error | 0.024 |  | 0.043 | 0.041 | 0.041 |
|  |  |  |  |  |  |
| Proportionate Change | 1.2% |  | -1.7% | 4.7% | 1.3% |
|  |  |  |  |  |  |
| Pre-announce mean outcome | 7403.19 |  | 6630.93 | 7278.24 | 8161.99 |
|  |  |  |  |  |  |
| **High Tax Soft Drink** |  |  |  |  |  |
| Point Estimate | 0.057 |  | 0.085 | 0.052 | 0.05 |
| Standard Error | 0.043 |  | 0.072 | 0.07 | 0.078 |
|  |  |  |  |  |  |
| Proportionate Effect | 5.9% |  | 8.9% | 5.3% | 5.1% |
|  |  |  |  |  |  |
| Pre-announce mean outcome | 3508.35 |  | 3478.83 | 3667.52 | 3408.17 |
|  |  |  |  |  |  |
| **Low Tax Soft Drink** |  |  |  |  |  |
| Point Estimate | -0.140* |  | -0.275* | -0.133 | -0.05 |
| Standard Error | 0.06 |  | 0.112 | 0.112 | 0.093 |
|  |  |  |  |  |  |
| Proportionate Change | -13.1%* |  | -24.0%* | -12.5% | -4.9% |
|  |  |  |  |  |  |
| Pre-announce mean outcome | 2669.15 |  | 2106.88 | 2465.28 | 3310.59 |
|  |  |  |  |  |  |
| **No Tax Soft Drink** |  |  |  |  |  |
| Point Estimate | -0.036 |  | -0.136 | -0.169 | 0.143 |
| Standard Error | 0.056 |  | 0.088 | 0.1 | 0.098 |
|  |  |  |  |  |  |
| Proportionate Change | -3.5% |  | -12.7% | -15.5% | 15.4% |
|  |  |  |  |  |  |
| Pre-announce mean outcome | 325.88 |  | 176.82 | 282.67 | 487.4 |
|  |  |  |  |  |  |
| **Sugar** |  |  |  |  |  |
| Point Estimate | 0.048 |  | 0.069 | 0.048 | 0.039 |
| Standard Error | 0.029 |  | 0.050 | 0.048 | 0.05 |
|  |  |  |  |  |  |
| Proportionate Change | 4.9% |  | 7.1% | 4.9% | 4.0% |
|  |  |  |  |  |  |
| Pre-announce mean outcome | 364.72 |  | 354.14 | 377.16 | 363.96 |
|  |  |  |  |  |  |
| **Number Households** | 2836 |  | 1120 | 963 | 1138 |
| **Number Observations** | 113044 |  | 36443 | 34010 | 42591 |
|  | |  |  |  |  |

Note: Proportionate Change = exp(point estimate) – 1. * p<0.05, **p<0.01, *** p<0.001
