# Supplementary material for: Evaluating the 2014 sugar-sweetened beverage tax in Chile: An observational study in urban areas
Source: PLoS Med. 2018 Jul 3;15(7):e1002596. doi: 10.1371/journal.pmed.1002596 (PMC6029775; doi:10.1371/journal.pmed.1002596)
Supplement: S5 Table — (DOCX) [file pmed.1002596.s015.docx]

**S5 Table**

**Regression analysis using alternate measures of pre-tax expenditure of soft drinks**

|  | **Pre-tax purchase of High Tax Soft Drinks  (based on total volume purchased)** | | | **Pre-tax purchase of High Tax Soft Drinks  (based on per capita volume purchased)** | | |
| --- | --- | --- | --- | --- | --- | --- |
| **All Soft Drink** | **Low** | **Middle** | **High** | **Low** | **Middle** | **High** |
| Point Estimate | 0.038 | -0.095* | -0.112*** | 0.036 | -0.073* | -0.137*** |
| Standard Error | 0.056 | 0.038 | 0.028 | 0.055 | 0.035 | 0.033 |
| **High Tax Soft Drink** |  |  |  |  |  |  |
| Point Estimate | -0.159 | -0.266** | -0.292*** | -0.097 | -0.243*** | -0.384*** |
| Standard Error | 0.099 | 0.071 | 0.053 | 0.094 | 0.068 | 0.064 |
| **Low Tax Soft Drink** |  |  |  |  |  |  |
| Point Estimate | 0.109 | -0.001 | -0.023 | 0.094 | 0.040 | -0.049 |
| Standard Error | 0.105 | 0.106 | 0.112 | 0.104 | 0.106 | 0.112 |
| **No Tax Soft Drink** |  |  |  |  |  |  |
| Point Estimate | -0.243*** | 0.041 | -0.13 | -0.232** | 0.033 | -0.128 |
| Standard Error | 0.089 | 0.091 | 0.099 | 0.087 | 0.091 | 0.101 |
| **Sugar** |  |  |  |  |  |  |
| Point Estimate | -0.099 | -0.169*** | -0.212*** | -0.077 | -0.139** | -0.270*** |
| Standard Error | 0.062 | 0.048 | 0.038 | 0.060 | 0.045 | 0.043 |
|  |  |  |  |  |  |  |
| **Number Households** | 1166 | 857 | 813 | 1165 | 864 | 807 |
| **Number Observations** | 37710 | 37654 | 37680 | 37641 | 37766 | 37637 |

Note: * p<0.05, **p<0.01, *** p<0.001
