# Supplementary material for: Evaluating the 2014 sugar-sweetened beverage tax in Chile: An observational study in urban areas
Source: PLoS Med. 2018 Jul 3;15(7):e1002596. doi: 10.1371/journal.pmed.1002596 (PMC6029775; doi:10.1371/journal.pmed.1002596)
Supplement: S6 Table — SES, socioeconomic status. (DOCX) [file pmed.1002596.s016.docx]

**S6 Table**

**Regression analysis using alternate measures of socioeconomic status group**

|  | **Education (alternate SES)** | | | | **Occupation (alternate SES)** | | | | **Alternate MCA-based index** | | |
| --- | --- | --- | --- | --- | --- | --- | --- | --- | --- | --- | --- |
| **All Soft Drink** | **None** | **Primary** | **Secondary** | **Post  Secondary** | **None** | **H'hold/ Unskilled** | **Skilled** | **Retired** | **Low MCA** | **Middle MCA** | **High MCA** |
| Point Estimate | -0.058 | -0.092 | -0.036 | -0.034 | -0.079 | -0.158* | -0.045 | -0.135* | -0.108** | -0.019 | -0.059 |
| Standard Error | 0.043 | 0.056 | 0.040 | 0.073 | 0.102 | 0.067 | 0.032 | 0.057 | 0.041 | 0.043 | 0.041 |
| **High Tax Soft Drink** |  |  |  |  |  |  |  |  |  |  |  |
| Point Estimate | -0.127 | -0.252** | -0.202** | -0.509*** | -0.196 | -0.152 | -0.251*** | -0.527*** | -0.148* | -0.154* | -0.429*** |
| Standard Error | 0.080 | 0.095 | 0.074 | 0.116 | 0.175 | 0.120 | 0.055 | 0.122 | 0.069 | 0.076 | 0.083 |
| **Low Tax Soft Drink** |  |  |  |  |  |  |  |  |  |  |  |
| Point Estimate | -0.061 | 0.178 | -0.045 | 0.213 | 0.082 | -0.044 | -0.060 | -0.004 | -0.155 | 0.147 | 0.102 |
| Standard Error | 0.117 | 0.146 | 0.101 | 0.153 | 0.229 | 0.177 | 0.079 | 0.166 | 0.109 | 0.111 | 0.101 |
| **No Tax Soft Drink** |  |  |  |  |  |  |  |  |  |  |  |
| Point Estimate | -0.032 | -0.119 | -0.156 | -0.214 | -0.195 | 0.053 | -0.215** | 0.019 | -0.069 | -0.006 | -0.255* |
| Standard Error | 0.102 | 0.126 | 0.083 | 0.158 | 0.231 | 0.163 | 0.067 | 0.147 | 0.088 | 0.094 | 0.101 |
| **Sugar** |  |  |  |  |  |  |  |  |  |  |  |
| Point Estimate | -0.112* | -0.165** | -0.120* | -0.336*** | -0.09 | -0.138 | -0.166*** | -0.342*** | -0.125** | -0.121* | -0.242*** |
| Standard Error | 0.056 | 0.062 | 0.048 | 0.076 | 0.111 | 0.082 | 0.0.36 | 0.08 | 0.047 | 0.051 | 0.052 |
|  |  |  |  |  |  |  |  |  |  |  |  |
| **Number Households** | 984 | 1016 | 1185 | 575 | 694 | 861 | 2151 | 491 | 1190 | 1319 | 1025 |
| **Number Observations** | 31809 | 25661 | 37962 | 17612 | 10663 | 17988 | 69800 | 14593 | 37523 | 38693 | 36828 |

Note: * p<0.05, **p<0.01, *** p<0.001
