# Supplementary material for: Evaluating the 2014 sugar-sweetened beverage tax in Chile: An observational study in urban areas
Source: PLoS Med. 2018 Jul 3;15(7):e1002596. doi: 10.1371/journal.pmed.1002596 (PMC6029775; doi:10.1371/journal.pmed.1002596)
Supplement: S7 Table — (DOCX) [file pmed.1002596.s017.docx]

**S7 Table**

**Regression analysis for the share of expenditure of each tax group in total expenditure on soft drinks**

|  | **All** | **SES** | | |
| --- | --- | --- | --- | --- |
| **High Tax Soft Drink** |  | **Low** | **Middle** | **High** |
| Point Estimate | -0.036*** | -0.014 | -0.045*** | -0.044*** |
| Standard Error | 0.005 | 0.009 | 0.009 | 0.008 |
|  |  |  |  |  |
| **Low Tax Soft Drink** |  |  |  |  |
| Point Estimate | 0.015** | -0.004 | 0.021** | 0.023** |
| Standard Error | 0.005 | 0.008 | 0.008 | 0.008 |
|  |  |  |  |  |
| **No Tax Soft Drink** |  |  |  |  |
| Point Estimate | -0.002 | -0.001 | 0.000 | -0.003 |
| Standard Error | 0.002 | 0.004 | 0.004 | 0.004 |
|  |  |  |  |  |
| **Number Households** | 2836 | 1120 | 963 | 1138 |
| **Number Observations** | 113044 | 36443 | 34010 | 42591 |

Note. * p<0.05, **p<0.01, *** p<0.001
