# Supplementary material for: Evaluating the 2014 sugar-sweetened beverage tax in Chile: An observational study in urban areas
Source: PLoS Med. 2018 Jul 3;15(7):e1002596. doi: 10.1371/journal.pmed.1002596 (PMC6029775; doi:10.1371/journal.pmed.1002596)
Supplement: S8 Table — BMI, body mass index. (DOCX) [file pmed.1002596.s018.docx]

**S8 Table**

**Regression analysis using alternate measures of body mass index group**

|  | **BMI (HH mean adult BMI)** | | | **Proportion Obese (>30 BMI)** | | | **Proportion Overweight (>25 BMI)** | | |
| --- | --- | --- | --- | --- | --- | --- | --- | --- | --- |
| **All Soft Drink** | **Low** | **Middle** | **High** | **Low** | **Middle** | **High** | **Low** | **Middle** | **High** |
| Point Estimate | -0.077 | -0.048 | -0.199** | -0.067* | -0.072* | -0.093* | -0.073 | -0.045 | -0.105* |
| Standard Error | 0.041 | 0.028 | 0.061 | 0.033 | 0.036 | 0.041 | 0.047 | 0.030 | 0.043 |
| **High Tax Soft Drink** |  |  |  |  |  |  |  |  |  |
| Point Estimate | -0.396*** | -0.159*** | -0.356** | -0.281*** | -0.213** | -0.286*** | -0.410*** | -0.159** | -0.257** |
| Standard Error | 0.082 | 0.056 | 0.111 | 0.066 | 0.075 | 0.084 | 0.097 | 0.059 | 0.084 |
| **Low Tax Soft Drink** |  |  |  |  |  |  |  |  |  |
| Point Estimate | 0.078 | 0.049 | -0.223 | 0.056 | 0.034 | -0.053 | 0.224 | 0.082 | -0.243 |
| Standard Error | 0.111 | 0.082 | 0.175 | 0.093 | 0.109 | 0.128 | 0.140 | 0.086 | 0.132 |
| **No Tax Soft Drink** |  |  |  |  |  |  |  |  |  |
| Point Estimate | -0.054 | -0.141* | -0.012 | -0.015 | -0.258** | -0.077 | -0.079 | -0.123 | -0.114 |
| Standard Error | 0.102 | 0.070 | 0.149 | 0.083 | 0.096 | 0.106 | 0.128 | 0.072 | 0.112 |
| **Sugar** |  |  |  |  |  |  |  |  |  |
| Point Estimate | -0.256*** | -0.105** | -0.279*** | -0.167*** | -0.169*** | -0.204*** | -0.270*** | -0.106** | -0.185*** |
| Standard Error | 0.053 | 0.037 | 0.074 | 0.042 | 0.050 | 0.056 | 0.065 | 0.038 | 0.056 |
|  |  |  |  |  |  |  |  |  |  |
| **Number Households** | 962 | 1705 | 735 | 1604 | 797 | 794 | 611 | 1536 | 933 |
| **Number Observations** | 33672 | 64104 | 15268 | 53636 | 30495 | 28388 | 22467 | 56394 | 27860 |

Note: * p<0.05, **p<0.01, *** p<0.001
