# Supplementary material for: Evaluating the 2014 sugar-sweetened beverage tax in Chile: An observational study in urban areas
Source: PLoS Med. 2018 Jul 3;15(7):e1002596. doi: 10.1371/journal.pmed.1002596 (PMC6029775; doi:10.1371/journal.pmed.1002596)
Supplement: S9 Table — AIC, Akaike Information Criterion. (DOCX) [file pmed.1002596.s019.docx]

**S9 Table**

**Sensitivity checks for the polynomial function using Akaike Information Criterion: Regression model for volume of soft drinks**

Note: AIC coefficient that is highlighted in yellow exhibits the minimum number amongst the models with different orders of polynomials. * p<0.05, **p<0.01, *** p<0.001
