# Supplementary material for: Evaluating the 2014 sugar-sweetened beverage tax in Chile: An observational study in urban areas
Source: PLoS Med. 2018 Jul 3;15(7):e1002596. doi: 10.1371/journal.pmed.1002596 (PMC6029775; doi:10.1371/journal.pmed.1002596)
Supplement: S10 Table — AIC, Akaike Information Criterion. (DOCX) [file pmed.1002596.s020.docx]

**S10 Table**

**Sensitivity checks for the polynomial function using Akaike Information Criterion: Regression model for price of soft drinks**

|  | **Posttax (Implementation)** | **AIC** |  | **Posttax (Announcement)** | **AIC** |
| --- | --- | --- | --- | --- | --- |
| **All Soft Drink** |  |  |  |  |  |
| Point Estimate (Fourth Order) | -0.010 | -31106.81 |  | 0.016** | -31112.07 |
| First Order | 0.002 | -31091.46 |  | 0.015*** | -31113.58 |
| Second Order | -0.001 | -31090.69 |  | 0.019*** | -31114.97 |
| Third Order | 0.000 | -31092.97 |  | 0.019*** | -31113.04 |
| Fifth Order | -0.010 | -31104.82 |  | 0.017** | -31112.29 |
| **High Tax Soft Drink** |  |  |  |  |  |
| Point Estimate (Fourth Order) | -0.008 | -13694.39 |  | 0.019* | -13699.35 |
| First Order | 0.017** | -13670.60 |  | 0.031*** | -13700.23 |
| Second Order | 0.007 | -13673.30 |  | 0.030*** | -13698.33 |
| Third Order | 0.010 | -13676.71 |  | 0.030*** | -13696.43 |
| Fifth Order | -0.007 | -13692.39 |  | 0.020* | -13698.14 |
| **Low Tax Soft Drink** |  |  |  |  |  |
| Point Estimate (Fourth Order) | -0.017* | -15290.12 |  | 0.013 | -15287.84 |
| First Order | -0.014* | -15292.58 |  | 0.001 | -15282.55 |
| Second Order | -0.011 | -15290.84 |  | 0.011 | -15291.42 |
| Third Order | -0.011 | -15289.34 |  | 0.011 | -15289.46 |
| Fifth Order | -0.016 | -15286.15 |  | 0.014 | -15288.52 |
| **No Tax Soft Drink** |  |  |  |  |  |
| Point Estimate (Fourth Order) | 0.017 | -2836.61 |  | 0.001 | -2835.64 |
| First Order | 0.026 | -2841.37 |  | 0.015 | -2836.21 |
| Second Order | 0.023 | -2839.45 |  | 0.002 | -2836.94 |
| Third Order | 0.021 | -2838.41 |  | 0.010 | -2836.92 |
| Fifth Order | 0.018 | -2834.61 |  | 0.000 | -2833.90 |

Note: AIC coefficient that is highlighted in yellow exhibits the minimum number amongst the models with different orders of polynomials. * p<0.05, **p<0.01, *** p<0.001
