# Supplementary material for: Evaluating the 2014 sugar-sweetened beverage tax in Chile: An observational study in urban areas
Source: PLoS Med. 2018 Jul 3;15(7):e1002596. doi: 10.1371/journal.pmed.1002596 (PMC6029775; doi:10.1371/journal.pmed.1002596)
Supplement: S11 Table — SES, socioeconomic status. (DOCX) [file pmed.1002596.s021.docx]

**S11 Table**

**Regression analysis for price paid by socioeconomic status**

|  |  |  |  |  |
| --- | --- | --- | --- | --- |
| ***A: Price Regression Model for High SES Group*** | |  |  |  |
|  | **All** | **High Tax** | **Low Tax** | **No Tax** |
| **Point Estimate** | 0.007 | 0.01 | -0.007 | 0.029 |
|  |  |  |  |  |
| **Proportionate Change** | 0.7% | 1% | -0.7% | 2.9% |
|  |  |  |  |  |
| **Number of Products** | 814 | 384 | 384 | 46 |
| **Number of Observations** | 32,059 | 16,130 | 13,761 | 2,168 |
| ***B: Price Regression Model for Middle SES Group*** | | |  |  |
|  | **All** | **High Tax** | **Low Tax** | **No Tax** |
| **Point Estimate** | 0.005 | -0.029** | -0.002 | -0.01 |
|  |  |  |  |  |
| **Proportionate Change** | 0.5% | -2.9%** | -0.2% | -1% |
|  |  |  |  |  |
| **Number of Products** | 816 | 386 | 384 | 46 |
| **Number of Observations** | 30,866 | 16,284 | 12,613 | 1,969 |
| ***C: Price Regression Model for Low SES Group*** | |  |  |  |
|  | **All** | **High Tax** | **Low Tax** | **No Tax** |
| **Point Estimate** | -0.007 | -0.021* | -0.023 | -0.012 |
|  |  |  |  |  |
| **Proportionate Change** | -0.7% | -2.1%* | -2.3% | -1.2% |
|  |  |  |  |  |
| **Number of Products** | 816 | 386 | 384 | 46 |
| **Number of Observations** | 30,548 | 16,861 | 11,839 | 1,848 |

Note: * p<0.05, **p<0.01, *** p<0.001
