# Supplementary material for: Evaluating the 2014 sugar-sweetened beverage tax in Chile: An observational study in urban areas
Source: PLoS Med. 2018 Jul 3;15(7):e1002596. doi: 10.1371/journal.pmed.1002596 (PMC6029775; doi:10.1371/journal.pmed.1002596)
Supplement: S12 Table — (DOCX) [file pmed.1002596.s022.docx]

**S12 Table**

**Regression analysis for frequency of shopping of soft drinks per month**

|  |  | **All** | **SES** | | |
| --- | --- | --- | --- | --- | --- |
| **High Tax Soft Drink** | |  | **Low** | **Middle** | **High** |
| Point Estimate | | -0.081*** | -0.053* | -0.108*** | -0.080*** |
| Standard Error | | 0.011 | 0.021 | 0.020 | 0.017 |
|  |  |  |  |  |  |
| Proportionate Change | | -7.8%*** | -5.2%* | -10.2% | -7.7%*** |
|  |  |  |  |  |  |
| **Low Tax Soft Drink** | |  |  |  |  |
| Point Estimate | | 0.015 | -0.024 | 0.010 | -0.051** |
| Standard Error | | 0.010 | 0.018 | 0.018 | 0.016 |
|  |  |  |  |  |  |
| Proportionate Change | | 1.5% | -2.4% | 1.0% | -5.0%** |
|  |  |  |  |  |  |
| **No Tax Soft Drink** | |  |  |  |  |
| Point Estimate | | -0.001 | 0.006 | 0.014 | -0.017 |
| Standard Error | | 0.006 | 0.010 | 0.011 | 0.010 |
|  |  |  |  |  |  |
| Proportionate Change | | -0.1% | 0.6% | 1.4% | -1.7% |
|  |  |  |  |  |  |
| **Number Households** | | 2836 | 1120 | 963 | 1138 |
| **Number Observations** | | 113044 | 36443 | 34010 | 42591 |

Note: Proportionate change = exp(point estimate) – 1. * p<0.05, **p<0.01, *** p<0.001
