# Supplementary material for: Evaluating the 2014 sugar-sweetened beverage tax in Chile: An observational study in urban areas
Source: PLoS Med. 2018 Jul 3;15(7):e1002596. doi: 10.1371/journal.pmed.1002596 (PMC6029775; doi:10.1371/journal.pmed.1002596)
Supplement: S13 Table — (DOCX) [file pmed.1002596.s023.docx]

**S13 Table**

**Regression analysis for use of price promotions**

|  |  | **All** | **SES** | | |
| --- | --- | --- | --- | --- | --- |
| **High Tax Soft Drink** | |  | **Low** | **Middle** | **High** |
| Point Estimate | | 0.006* | 0.002 | 0.003 | 0.011** |
| Standard Error | | 0.002 | 0.003 | 0.004 | 0.004 |
|  |  |  |  |  |  |
| Proportionate Change | | 0.6%* | 0.2% | 0.3% | 1.1%** |
|  |  |  |  |  |  |
| **Low Tax Soft Drink** | |  |  |  |  |
| Point Estimate | | 0.009*** | 0.013** | 0.006 | 0.008 |
| Standard Error | | 0.002 | 0.004 | 0.004 | 0.004 |
|  |  |  |  |  |  |
| Proportionate Change | | 0.9%*** | 1.3%** | 0.6% | 0.8% |
|  |  |  |  |  |  |
| **No Tax Soft Drink** | |  |  |  |  |
| Point Estimate | | -0.001 | -0.001 | 0.004 | -0.006* |
| Standard Error | | 0.001 | 0.002 | 0.002 | 0.003 |
|  |  |  |  |  |  |
| Proportionate Change | | -0.1% | -0.1% | 0.4% | -0.6%* |
|  |  |  |  |  |  |
| **Number Households** | | 2836 | 1120 | 963 | 1138 |
| **Number Observations** | | 113044 | 36443 | 34010 | 42591 |

Note: Proportionate change = exp(point estimate) – 1. * p<0.05, **p<0.01, *** p<0.001
