# Supplementary material for: Evaluating the 2014 sugar-sweetened beverage tax in Chile: An observational study in urban areas
Source: PLoS Med. 2018 Jul 3;15(7):e1002596. doi: 10.1371/journal.pmed.1002596 (PMC6029775; doi:10.1371/journal.pmed.1002596)
Supplement: S14 Table — Volume regression results by product type ([A] ready-to-drink and concentrated; and [B] carbonated and noncarbonated) and by type of retailer ([C] major store chain and local or private store). (DOCX) [file pmed.1002596.s024.docx]

**S14 Table**

**Volume regression results by product type (A. ready-to-drink and concentrated; and B. carbonated and non-carbonated) and by type of retailer (C. major store chain and local or private store)**

| ***A: Ready to drink and Concentrated*** | |  |  |  |  |  |  |  |
| --- | --- | --- | --- | --- | --- | --- | --- | --- |
|  | **All Soft drinks** | | **High Tax** | | **Low Tax** | | **No Tax** | |
|  | **Ready to drink** | **Concentrated** | **Ready to drink** | **Concentrated** | **Ready to drink** | **Concentrated** | **Ready to drink** | **Concentrated** |
| Point estimate | -0.092** | -0.043 | -0.242*** | -0.048** | 0.124* | -0.072 | -0.109* | - |
| Standard Error | (0.028) | (0.067) | (0.043) | (0.017) | (0.058) | (0.068) | (0.054) | - |
|  |  |  |  |  |  |  |  |  |
| Proportionate Change | -8.79% | -4.21% | -21.49% | -4.69% | 13.20% | -6.95% | -10.33% | - |
|  |  |  |  |  |  |  |  |  |
| Pre-tax mean outcome (ml) | 5126.5 | 2253.6 | 3528.5 | 32.1 | 336.0 | 1871.6 | 32.1 | - |
| Number of households | 2,836 | 2,836 | 2,836 | 2,836 | 2,836 | 2,836 | 2,836 | - |
| Number of observations | 112,519 | 112,519 | 112,519 | 112,519 | 112,519 | 112,519 | 112,519 | - |
| ***B: Carbonated and Non-carbonated*** | |  |  |  |  |  |  |  |
|  | **All Soft drinks** | | **High Tax** | | **Low Tax** | | **No Tax** | |
|  | **Carbonated** | **Non-carbonated** | **Carbonated** | **Non-carbonated** | **Carbonated** | **Non-carbonated** | **Carbonated** | **Non-carbonated** |
| Point estimate | -0.188*** | -0.031 | -0.319*** | -0.130* | 0.059 | -0.025 | - | -0.109* |
| Standard Error | (0.044) | (0.046) | (0.052) | (0.060) | (0.046) | (0.066) | - | (0.054) |
|  |  |  |  |  |  |  |  |  |
| Proportionate Change | -17.14% | -3.05% | -27.31% | -12.19% | 6.08% | -2.47% | - | -10.33% |
|  |  |  |  |  |  |  |  |  |
| Pre-tax mean outcome | 3765.1 | 3615.0 | 2993.2 | 567.3 | 492.7 | 2147.5 | - | 336.0 |
| Number of households | 2,836 | 2,836 | 2,836 | 2,836 | 2,836 | 2,836 | - | 2,836 |
| Number of observations | 112,519 | 112,519 | 112,519 | 112,519 | 112,519 | 112,519 | - | 112,519 |
| ***C: Major store chain and local/private stores*** | |  |  |  |  |  |  |  |
|  | **All Soft drinks** | | **High Tax** | | **Low Tax** | | **No Tax** | |
|  | **Major store** | **Local store** | **Major store** | **Local store** | **Major store** | **Local store** | **Major store** | **Local store** |
| Point estimate | -0.215*** | -0.029 | -0.344*** | -0.168** | -0.096 | 0.058 | -0.117* | -0.025 |
| Standard Error | (0.052) | (0.053) | (0.057) | (0.055) | (0.061) | (0.059) | (0.046) | (0.037) |
|  |  |  |  |  |  |  |  |  |
| Proportionate Change | -19.35% | -2.86% | -29.11% | -15.46% | -9.15% | 5.97% | -11.04% | -2.47% |
|  |  |  |  |  |  |  |  |  |
| Pre-tax mean outcome | 3708.1 | 3672.0 | 1404.3 | 2156.3 | 1645.0 | 995.1 | 241.1 | 94.8 |
| Number of households | 2,836 | 2,836 | 2,836 | 2,836 | 2,836 | 2,836 | 2,836 | 2,836 |
| Number of observations | 112,519 | 112,519 | 112,519 | 112,519 | 112,519 | 112,519 | 112,519 | 112,519 |

Note: Proportionate change = exp(point estimate) - 1. * p<0.05, **p<0.01, *** p<0.001
