# Supplementary material for: Evaluating the 2014 sugar-sweetened beverage tax in Chile: An observational study in urban areas
Source: PLoS Med. 2018 Jul 3;15(7):e1002596. doi: 10.1371/journal.pmed.1002596 (PMC6029775; doi:10.1371/journal.pmed.1002596)
Supplement: S15 Table — (DOCX) [file pmed.1002596.s025.docx]

**S15 Table**

**Regression analysis for volume of soft drinks purchased by further disaggregated sub-group**

| ***A: SES by Pre-Tax Volume*** | |  |  |  |  |  |  |  |  |  |
| --- | --- | --- | --- | --- | --- | --- | --- | --- | --- | --- |
|  | **All** | **Low SES** | | | **Mid SES** | | | **High SES** | | |
|  |  | **Low Vol** | **Mid Vol** | **High Vol** | **Low Vol** | **Mid Vol** | **High Vol** | **Low Vol** | **Mid Vol** | **High Vol** |
| **All Soft drinks** | -0.060* | 0.103 | -0.107 | -0.095 | -0.009 | -0.107 | -0.076 | 0.052 | -0.067 | -0.156*** |
| **High Tax** | -0.244*** | -0.063 | -0.159 | -0.137 | -0.001 | -0.211 | -0.295*** | -0.328* | -0.386*** | -0.419*** |
| **Low Tax** | 0.03 | 0.014 | -0.229 | -0.374 | 0.152 | 0.012 | 0.425* | 0.117 | 0.2 | -0.121 |
| **No Tax** | -0.105 | -0.21 | 0.121 | -0.137 | -0.161 | 0.178 | 0.17 | -0.276 | -0.166 | -0.370* |
| **Sugar** | -0.164*** | -0.019 | -0.116 | -0.12 | -0.017 | -0.182* | -0.218** | -0.205* | -0.192* | -0.284*** |
| **No. of Households** | 2836 | 482 | 332 | 306 | 365 | 310 | 288 | 437 | 318 | 297 |
| **No. of Observations** | 113044 | 12338 | 12383 | 38355 | 10147 | 11666 | 12197 | 15225 | 13605 | 13761 |
| ***B: SES by BMI*** |  |  |  |  |  |  |  |  |  |  |
|  | **All** | **Low SES** | | | **Mid SES** | | | **High SES** | | |
|  |  | **Low BMI** | **Mid BMI** | **High BMI** | **Low BMI** | **Mid BMI** | **High BMI** | **Low BMI** | **Mid BMI** | **High BMI** |
| **All** | -0.060* | -0.139 | -0.013 | -0.067 | 0.043 | -0.105* | -0.259* | -0.095 | -0.021 | -0.323* |
| **High Tax** | -0.244*** | -0.241 | -0.053 | -0.253 | -0.28 | -0.113 | -0.426* | -0.519*** | -0.289** | -0.368 |
| **Low Tax** | 0.03 | 0.237 | -0.366* | -0.115 | 0.362 | 0.202 | -0.133 | -0.136 | 0.308* | -0.653 |
| **No Tax** | -0.105 | -0.232 | -0.094 | 0.151 | 0.667** | -0.065 | -0.175 | -0.313* | -0.248* | -0.041 |
| **Sugar** | -0.164*** | -0.166 | -0.034 | -0.200* | -0.221* | -0.1 | -0.331* | -0.308*** | -0.169** | -0.281 |
| **No. of Households** | 2836 | 294 | 674 | 325 | 292 | 623 | 227 | 442 | 588 | 228 |
| **No. of Observations** | 113044 | 8260 | 21099 | 7084 | 8554 | 20860 | 4596 | 16858 | 22145 | 3588 |
| ***C: Pre-tax volume by BMI*** |  |  |  |  |  |  |  |  |  |  |
|  | **All** | **Low Vol** | | | **Mid Vol** | | | **High Vol** | | |
|  |  | **Low BMI** | **Mid BMI** | **High BMI** | **Low BMI** | **Mid BMI** | **High BMI** | **Low BMI** | **Mid BMI** | **High BMI** |
| **All** | -0.060* | 0.032 | 0.022 | -0.28 | -0.151* | -0.063 | -0.16 | -0.119* | -0.105** | -0.182* |
| **High Tax** | -0.244*** | -0.214 | -0.107 | -0.589* | -0.511*** | -0.168 | -0.224 | -0.473*** | -0.194** | -0.316* |
| **Low Tax** | 0.03 | 0.25 | 0.035 | -0.125 | 0.015 | 0.07 | -0.303 | -0.053 | 0.028 | -0.205 |
| **No Tax** | -0.105 | -0.194 | -0.214 | -0.490* | 0.122 | -0.074 | 0.366 | -0.079 | -0.162 | -0.062 |
| **Sugar** | -0.164*** | -0.155 | -0.049 | -0.419* | -0.291** | -0.116 | -0.179 | -0.324*** | -0.143** | -0.267** |
| **No. of Households** | 2836 | 419 | 692 | 325 | 281 | 526 | 223 | 262 | 487 | 186 |
| **No. of Observations** | 113044 | 12041 | 21692 | 3977 | 10873 | 21579 | 5202 | 10758 | 20833 | 6089 |

Note: Proportionate effect = exp(point estimate) – 1. * p<0.05, **p<0.01, *** p<0.001
