# Supplementary material for: Evaluating the 2014 sugar-sweetened beverage tax in Chile: An observational study in urban areas
Source: PLoS Med. 2018 Jul 3;15(7):e1002596. doi: 10.1371/journal.pmed.1002596 (PMC6029775; doi:10.1371/journal.pmed.1002596)
Supplement: S1 Text — DiD, difference-in-difference. (DOCX) [file pmed.1002596.s026.docx]

**S1 Text**

**A quasi-difference-in-differences approach**

A difference-in-differences (DiD) approach was also attempted as an alternate analysis. For each household, purchasing outcomes in the pre-tax period and the post-tax period were compared between the implementation year (2014/15) and the previous calendar years (2012-14).^[[1]](#footnote-1)^ This approach assumes that the trend of household beverage purchases is comparable over years where there is no SSB tax. The use of the same households as their own comparison group, though in a different time period, is a departure from the standard DiD approach that uses distinct groups.

For each household and “year” (12-month period starting from September), we calculated the total volume of SSB from non-alcohol beverages purchased. We then constructed a biannual panel dataset. This means that for each household we calculated SSB purchases in the following periods:

1. April 2013-September 2013 (Period 1, orange)
2. October 2013-March 2014 (Period 2, yellow)
3. April 2014-September 2014 (Period 3, green)
4. October 2014-March 2015 (Period 4, blue)

|  |  |  |  |  |  |  |  |  |  |  |  |  |  |
| --- | --- | --- | --- | --- | --- | --- | --- | --- | --- | --- | --- | --- | --- |
| 2012 |  | 1 | 2 | 3 | 4 | 5 | 6 | 7 | 8 | 9 | 10 | 11 | 12 |
| 2013 |  | 1 | 2 | 3 | 4 | 5 | 6 | 7 | 8 | 9 | 10 | 11 | 12 |
| 2014 |  | 1 | 2 | 3 | 4 | 5 | 6 | 7 | 8 | 9 | 10 | 11 | 12 |
| 2015 |  | 1 | 2 | 3 | 4 | 5 | 6 | 7 | 8 | 9 | 10 | 11 | 12 |
|  |  |  |  |  |  |  |  |  |  |  |  |  |  |

Since the policy was implemented in October 2014, we first calculated the difference between the volume of SSB purchased in Period 3 (green) and that in Period 4 (blue). This gives a generic pre-post difference of SSB purchasing in the implementation year. Next, we assume that the trends of SSB purchasing (after controlling for year-specific factors) are comparable across different years (in the absence of the tax implementation). We calculate the difference between the volume of SSB purchased in Period 1 (orange) and that in Period 2 (yellow). This second difference will be used as the counterfactual, i.e. what would have happened, had the policy not been implemented in October 2014.

The DiD analysis was conducted based on a regression framework. Let $Y_{it}$ be the volume of SSB purchased for household *i* at period *t*. “*Post*” is a dummy variable taking 1 if the values come from Period 2 and Period 4, and taking 0 otherwise. “*ImpYr*” is also a dummy variable taking 1 if the values are from Period 3 and Period 4 i.e. implementation year, and 0 otherwise. We estimate the following linear fixed effects model:

$$Y_{it}=\alpha_{i}+\beta{Post}_{it}+\gamma{ImpYr}_{it}+\tau\left[ {Post}_{it}\times{ImpYr}_{it} \right]+\mathbf{z}_{\mathrm{it}}^{\mathbf{'}}\boldsymbol{\theta+}\varepsilon_{it}.$$

The variable of interest – change in the volume of soft drinks purchased - is represented by the parameter $\tau$. Fixed effects are included in the model in order to address the fact that the purchasing patterns are heterogeneous across households (but are assumed not to be time varying). Household level cluster robust standard errors were estimated. Most of the household characteristics are time invariant factors. However, if some characteristics vary over time e.g. temperature and macroeconomic factors (regional income level), we include those variables as control variables (as represented by vector $\mathbf{z}_{\mathrm{it}}^{'}$).

The above analysis uses a two-period model, but the main model exploits the longitudinal nature of the data. The regression model is then as follows, using additional time points *t* (e.g. month/week):

$Y_{\mathrm{it}}=\alpha_{i}+\tau\left[ {Post}_{it}\times{ImpYr}_{it} \right]+\mathbf{z}_{\mathrm{it}}\boldsymbol{'\theta}+\delta_{t}+\varepsilon_{\mathrm{it}}$

The variable $\delta_{t}$ shows the time period dummy e.g. month dummy to flexibly capture any seasonal effects. The change in the volume of soft drinks purchased is again represented by the parameter $\tau$.

A key assumption for identification of the parameter $\tau$ here is the so-called “parallel trend assumption”, i.e. in the absence of the tax, the trends (not levels) of conditional purchases of SSBs are the same between year 2013/4 and 2014/5. This assumption cannot be directly tested. However, we indirectly checked the validity of the assumption by assessing the trend of purchasing from October 2011 to September 2014.

**Reference**

1. Colchero MA, Popkin BM, Rivera JA, Ng SW. Beverage purchases from stores in Mexico under the excise tax on sugar sweetened beverages: observational study. BMJ. 2016;352:h6704

1. Colchero et al. (2016) used a similar approach in their evaluation of the Mexican soda tax – i.e. comparing the (contemporary) trend of purchasing with the trend that is predicted from the past trend.. [↑](#footnote-ref-1)
