# Supplementary material for: Evaluating the 2014 sugar-sweetened beverage tax in Chile: An observational study in urban areas
Source: PLoS Med. 2018 Jul 3;15(7):e1002596. doi: 10.1371/journal.pmed.1002596 (PMC6029775; doi:10.1371/journal.pmed.1002596)
Supplement: S2 Text — SES, socioeconomic status. (DOCX) [file pmed.1002596.s027.docx]

**S2 Text**

**Alternative socioeconomic status measures**

A socioeconomic status (SES) index was built using Multiple Correspondence Analysis (MCA) at household level using the ‘mca’ command in Stata. The variables included in the analysis were the occupation and education status of the household head and his/her partner, car ownership, number of bedrooms and number of bathrooms per household. Occupation and the highest educational level attained were measured using 12 categories (see below). The number of cars, bedrooms and bathrooms were divided by the number of household members, and then categorized in quintiles.

After running the MCA analysis, a score was estimated using the first principal component or dimension as weight for each household variable. The score was categorized in tertiles. The index showed a high level of correspondence with the original SES classification provided by Kantar (S1 Fig).

For a detailed description of MCA and other multivariate methods to build composite socioeconomic measures, see Vyas and Kumaranayake[1] and Nardo et al. [2].

Occupational categories

| 1 | Occasional unskilled work |
| --- | --- |
| 2 | Unskilled work |
| 3 | Lower managerial, administrative and professional occupations, intermediate occupations, small employers and own account workers, lower supervisory, craft and related occupations, semi routine and routine occupations. |
| 4 | High managers and administrative occupations in medium or small size firms, or higher professional occupations (doctor, barrister, architect, economist, engineer, agronomist) |
| 5 | High managers and administrative occupations in large firms |
| 6 | Homemaker, inactive |
| 7 | Homemaker, unemployed |
| 8 | Unemployed |
| 9 | Pensioner |
| 10 | Disabled, inactive |
| 11 | Inactive, income based on investments only |
| 12 | Student, inactive |

Education categories

| 1 | No formal education |
| --- | --- |
| 2 | Adult literacy without formal education |
| 3 | Incomplete primary education |
| 4 | Complete primary education |
| 5 | Incomplete secondary education, scientific-humanistic studies |
| 6 | Complete secondary education, scientific humanistic studies |
| 7 | Incomplete secondary education, technical-professional studies |
| 8 | Complete secondary education, technical-professional studies |
| 9 | Incomplete higher education |
| 10 | Complete higher education |
| 11 | Incomplete postgraduate education |
| 12 | Complete postgraduate education |

Assets and househould characteristics

| 1 | Number of cars |
| --- | --- |
| 2 | Number of bathrooms |
| 3 | Number of bedrooms |

**Reference**

1. Vyas S, Kumaranayake L. Constructing socio-economic status indices: how to use principal components analysis. Health Policy Plan. 2006;21:459–68.

2. Nardo M, Saisana M, Saltelli A, Tarantola S, Hoffmann A, Giovannini E. Handbook on Constructing Composite Indicators: Methodology and User Guide. OECD publishing; 2008.
