# Supplementary material for: Evaluating the 2014 sugar-sweetened beverage tax in Chile: An observational study in urban areas
Source: PLoS Med. 2018 Jul 3;15(7):e1002596. doi: 10.1371/journal.pmed.1002596 (PMC6029775; doi:10.1371/journal.pmed.1002596)
Supplement: S3 Text — IABA, Impuesto Adicional a las Bebidas Analcohólicas. (DOCX) [file pmed.1002596.s028.docx]

**S3 Text**

**Trend of keyword search of the IABA tax policy in the Internet**

The assumption that potential informational responses due to information transfer is explored using public available data. If any educational or communicational campaign was adopted during the period of the tax announcement or implementation to expect that changes in the web-based searches would be expected. Previous experiences analyzing web-based searches on key topics had been proven useful to monitor relevant informational shocks in the health sector [1].

To explore potential impacts of the tax announcement and implementation on consumer’s information seeking behaviors, we used Google Trends [2] search engine for “bebida” and related terms, Spanish equivalents for soda, fizzy drink or beverage for Chile in the period from 1/1/13 to 31/12/15 (S5 Fig). No breaks in the long-run tendency of internet search for the terms was observed close to the announcement (gray dotted vertical line) or implementation of the tax (green dotted vertical line). Vertical axis represents a metric of the popularity of the search term on a particular region and time period. More information on the metric could be found elsewhere [3].

A composite search strategy including the words (*gaseosa* AND *impuesto)* (in blue line), (*bebida* AND *impuesto)* (in red line) and (*Impuesto* AND *adicional* AND *bebidas* AND *analcohólicas*) (in yellow line) are shown in S6 Fig. There are seasonal peaks in March and April, however, the peak is pronounced more pronounced in 2014, when the tax policy was announced. A small peak is also observed in the week of September 28^th^ to October 4^th^ 2014, just before and after the implementation of the tax policy.

**Reference**

1. Nuti SV, Wayda B, Ranasinghe I, Wang S, Dreyer RP, Chen SI, et al. (2014) The Use of Google Trends in Health Care Research: A Systematic Review. PLoS ONE 9(10): e109583. <https://doi.org/10.1371/journal.pone.0109583>
2. Google (2018) Google Trends. Accessed 2018 January 23.
3. Google (2018) Google Trends. How Trends data is adjusted https://support.google.com/trends/answer/4365533?hl=en&ref_topic=6248052. Accessed 2018 January 23.
