# Supplementary material for: Evaluating the 2014 sugar-sweetened beverage tax in Chile: An observational study in urban areas
Source: PLoS Med. 2018 Jul 3;15(7):e1002596. doi: 10.1371/journal.pmed.1002596 (PMC6029775; doi:10.1371/journal.pmed.1002596)
Supplement: S1 STROBE — (DOCX) [file pmed.1002596.s029.docx]

**S1 STROBE**

STROBE Statement—checklist of items that should be included in reports of observational studies

|  | Item No | Recommendation |
| --- | --- | --- |
| **Title and abstract** | 1 | (*a*) Indicate the study’s design with a commonly used term in the title or the abstract  **Indicated in Abstract, “Methods and Findings” section** |
|  |  | (*b*) Provide in the abstract an informative and balanced summary of what was done and what was found  **Done in Abstract.** |
| Introduction | | |
| Background/rationale | 2 | Explain the scientific background and rationale for the investigation being reported  **Done in Introduction, paragraph 1-5.** |
| Objectives | 3 | State specific objectives, including any prespecified hypotheses  **Done in Introduction, paragraph 6.** |
| Methods | | |
| Study design | 4 | Present key elements of study design early in the paper  **Presented in Abstract, “Methods and Finings”; and in Methods, “Analytic methods” sub-section.** |
| Setting | 5 | Describe the setting, locations, and relevant dates, including periods of recruitment, exposure, follow-up, and data collection  **Described in Methods, “Data” sub-section.** |
| Participants | 6 | (*a*) *Cohort study*—Give the eligibility criteria, and the sources and methods of selection of participants. Describe methods of follow-up  **Done in Methods, “Data” sub-section.**  *Case-control study*—Give the eligibility criteria, and the sources and methods of case ascertainment and control selection. Give the rationale for the choice of cases and controls  *Cross-sectional study*—Give the eligibility criteria, and the sources and methods of selection of participants |
|  |  | (*b*) *Cohort study*—For matched studies, give matching criteria and number of exposed and unexposed  *Case-control study*—For matched studies, give matching criteria and the number of controls per case |
| Variables | 7 | Clearly define all outcomes, exposures, predictors, potential confounders, and effect modifiers. Give diagnostic criteria, if applicable  **Done in Methods, “Analytic methods” sub-section, paragraph 3 and 4.** |
| Data sources/ measurement | 8* | For each variable of interest, give sources of data and details of methods of assessment (measurement). Describe comparability of assessment methods if there is more than one group  **Done in Methods, “Data” sub-section.** |
| Bias | 9 | Describe any efforts to address potential sources of bias  **Done in Methods, “Data” sub-section, paragraph 5-6; also in “Analytic methods” sub-section, paragraph 3 - 5.** |
| Study size | 10 | Explain how the study size was arrived at  **Done in Methods, “Data” sub-section, paragraph 1.** |
| Quantitative variables | 11 | Explain how quantitative variables were handled in the analyses. If applicable, describe which groupings were chosen and why  **Done in Methods, “Analytic methods” sub-section, paragraph 3 and 4.** |
| Statistical methods | 12 | 1. Describe all statistical methods, including those used to control for confounding   **Done in Methods, “Analytic methods” sub-section, paragraph 3 and 4.** |
|  |  | (*b*) Describe any methods used to examine subgroups and interactions  **Done in Methods, “Outcome variables and sub-sample analyses” sub-section.** |
|  |  | (*c*) Explain how missing data were addressed  **Explained in Methods, “Data” sub-section, paragraph 5-6.** |
|  |  | (*d*) *Cohort study*—If applicable, explain how loss to follow-up was addressed  **Explained in Methods, “Data” sub-section, paragraph 5-6.**  *Case-control study*—If applicable, explain how matching of cases and controls was addressed  *Cross-sectional study*—If applicable, describe analytical methods taking account of sampling strategy |
|  |  | (*e*) Describe any sensitivity analyses  **Done in S1 Table, S9 Table and S10 Table; and described in Results, “Volume of soft drinks purchased” sub-section.** |

Continued on next page

| Results | | |
| --- | --- | --- |
| Participants | 13* | (a) Report numbers of individuals at each stage of study—eg numbers potentially eligible, examined for eligibility, confirmed eligible, included in the study, completing follow-up, and analysed  **Explained in Methods, “Data” sub-section, paragraph 1, 5-6.** |
|  |  | (b) Give reasons for non-participation at each stage  **Explained in Methods, “Data” sub-section, paragraph 5-6.** |
|  |  | (c) Consider use of a flow diagram  **Considered, but decided not to use one in this observational data analysis.** |
| Descriptive data | 14* | (a) Give characteristics of study participants (eg demographic, clinical, social) and information on exposures and potential confounders  **Done in Table 1, in S3 Table, and in Results, paragraph 1.** |
|  |  | (b) Indicate number of participants with missing data for each variable of interest  **Done in Table 1 and S3 Table** |
|  |  | (c) *Cohort study*—Summarise follow-up time (eg, average and total amount)  **Explained in Methods, “Data” sub-section** |
| Outcome data | 15* | *Cohort study*—Report numbers of outcome events or summary measures over time  **Done in Table 1, Fig 1, and S3 Table.** |
|  |  | *Case-control study—*Report numbers in each exposure category, or summary measures of exposure |
|  |  | *Cross-sectional study—*Report numbers of outcome events or summary measures |
| Main results | 16 | (*a*) Give unadjusted estimates and, if applicable, confounder-adjusted estimates and their precision (eg, 95% confidence interval). Make clear which confounders were adjusted for and why they were included  **Done in Figure 1 and Table 2. A description of confounders and statistical adjustments are explained in Methods, “Analytic methods” sub-section, paragraph 3 - 5***.* |
|  |  | (*b*) Report category boundaries when continuous variables were categorized  **Not applicable.** |
|  |  | (*c*) If relevant, consider translating estimates of relative risk into absolute risk for a meaningful time period  **Done in Table 2 (Proportionate change).** |
| Other analyses | 17 | Report other analyses done—eg analyses of subgroups and interactions, and sensitivity analyses  **Done in Result, “Volume of soft drinks purchased” sub-section, paragraph 6-7; “Paid price” sub-section, paragraph 3; “Shopping pattern” sub-section, paragraph 3.** |
| Discussion | | |
| Key results | 18 | Summarise key results with reference to study objectives  **Done in Discussion, paragraph 1.** |
| Limitations | 19 | Discuss limitations of the study, taking into account sources of potential bias or imprecision. Discuss both direction and magnitude of any potential bias  **Done in Discussion, “Strength and limitations” sub-section***.* |
| Interpretation | 20 | Give a cautious overall interpretation of results considering objectives, limitations, multiplicity of analyses, results from similar studies, and other relevant evidence  **Done in Discussion, “Strength and limitations” sub-section***.* |
| Generalisability | 21 | Discuss the generalisability (external validity) of the study results  **Done in Discussion, “Strength and limitations” sub-section***.* |
| Other information | | |
| Funding | 22 | Give the source of funding and the role of the funders for the present study and, if applicable, for the original study on which the present article is based  **Done in the online submission process.** |

*Give information separately for cases and controls in case-control studies and, if applicable, for exposed and unexposed groups in cohort and cross-sectional studies.

**Note:** An Explanation and Elaboration article discusses each checklist item and gives methodological background and published examples of transparent reporting. The STROBE checklist is best used in conjunction with this article (freely available on the Web sites of PLoS Medicine at http://www.plosmedicine.org/, Annals of Internal Medicine at http://www.annals.org/, and Epidemiology at http://www.epidem.com/). Information on the STROBE Initiative is available at www.strobe-statement.org.
